# High-resolution microbiome analysis enabled by linking of 16S rRNA gene sequences with adjacent genomic contexts

**DOI:** 10.1099/mgen.0.000624

**Published:** 2021-09-02

**Authors:** Žana Kapustina, Justina Medžiūnė, Gediminas Alzbutas, Irmantas Rokaitis, Karolis Matjošaitis, Gytis Mackevičius, Simona Žeimytė, Laurynas Karpus, Arvydas Lubys

**Affiliations:** ^1^​ Thermo Fisher Scientific Baltics, V. A. Graičiūno str. 8, Vilnius 02241, Lithuania; ^2^​ Institute of Biosciences, Life Sciences Center, Vilnius University, Saulėtekio al. 7, Vilnius 10257, Lithuania; ^3^​ Institute of Chemistry, Faculty of Chemistry and Geosciences, Vilnius University, Naugarduko str. 24, Vilnius 03225, Lithuania; ^4^​ Biomatter Designs, Žirmūnų str. 139A, Vilnius 09120, Lithuania

**Keywords:** 16S rRNA, high-throughput microbiome profiling, semi-targeted sequencing, targeted DNA sequencing

## Abstract

Sequence-based characterization of bacterial communities has long been a hostage of limitations of both 16S rRNA gene and whole metagenome sequencing. Neither approach is universally applicable, and the main efforts to resolve constraints have been devoted to improvement of computational prediction tools. Here, we present semi-targeted 16S rRNA sequencing (st16S-seq), a method designed for sequencing V1–V2 regions of the 16S rRNA gene along with the genomic locus upstream of the gene. By *in silico* analysis of 13 570 bacterial genome assemblies, we show that genome-linked 16S rRNA sequencing is superior to individual hypervariable regions or full-length gene sequences in terms of classification accuracy and identification of gene copy numbers. Using mock communities and soil samples we experimentally validate st16S-seq and benchmark it against the established microbial classification techniques. We show that st16S-seq delivers accurate estimation of 16S rRNA gene copy numbers, enables taxonomic resolution at the species level and closely approximates community structures obtainable by whole metagenome sequencing.

## Data Summary

The raw sequencing data were deposited in the NCBI SRA repository under BioProject accession number PRJNA695397. The custom code used for st16S-seq data analysis will be available at GitHub before publication.

**Impact Statement**

The 16S rRNA gene is widely used to differentiate operational taxonomic units for the profiling of microbial communities. High-throughput sequencing of 16S rRNA amplicons led to rapid growth of available gene sequence data, which to this day outnumber complete genome assemblies. Despite its versatility, intragenomic heterogeneity of 16S rRNA gene copies impairs classification accuracy as well as quantitative representation of microbial communities. Long-read sequencing technologies and improved computational prediction tools are being offered to increase the accuracy of 16S rRNA gene sequence analysis, but new experimental techniques which would substantially increase the informativeness of targeted sequencing are needed to achieve truly unbiased characterization. We show that looking beyond the 16S rRNA gene is a beneficial strategy and suggest a library preparation method that can capture any unknown sequence near a defined target site. Semi-targeted 16S rRNA gene sequencing directly links each 16S rRNA gene copy with an adjacent genomic locus upstream of the gene and enables highly accurate classification and unambiguous quantification of taxa. This technique opens up a new perspective for highly accurate characterization of microbial communities by next-generation sequencing at a cost of targeted sequencing.

## Introduction

High-throughput amplicon sequencing has fuelled microbiome research by providing tools for culture- and cloning-free analysis of bacterial phylogenetic marker genes, among which the 16S rRNA gene, consisting of nine hypervariable regions (V1–V9) flanked by conserved sequences, is the molecular chronometer of choice [1] for the vast majority of microbial diversity studies, including global initiatives such as the Earth Microbiome Project [2] and the Human Microbiome Project [3].

Typically, 16S rRNA gene sequences, corresponding to a single or multiple amplified hypervariable regions, are clustered based on similarity to obtain operational taxonomic units (OTUs). Representative OTU sequences are then compared to reference databases to infer taxonomy with an assumed identity threshold for species-level identification of 97% or in the range of 97–99% [4–6]. Nevertheless, consensus sequences cannot ensure reliable classification or discriminate between closely related species as 16S rRNA gene sequences may differ by SNPs, which may not be located within the hypervariable regions [7]. On the other hand, sequence variation might be often characteristic of different 16S rRNA gene copies within a single genome [8]. Base-level analysis of the full-length 16S rRNA gene is now possible due to the emergence of long-read sequencing technologies, but sequencing errors and ambiguities related to the variability of 16S rRNA gene copy numbers still pose a significant challenge to accurate interpretation of microbial diversity and quantitative estimates of the proportions of taxa.

Microbial profiling data able to achieve species and strain-level resolution have great potential to uncover the recent evolution of microbial populations, shed light on functional differences between communities [9], and provide insights on host colonization processes [10] and transmission patterns between hosts [11]. Unveiling strain-level variation across communities is generally performed by whole metagenome sequencing, which can capture genome-wide patterns of genetic polymorphism. Comprehensive data come with a cost of much deeper sequencing required to detect a reasonable number of community members. Moreover, the choice of analytical strategy in this case depends on the sample nature; for example, reference-based methods work well for species from the human microbiome which are well represented in databases, but are not effective for environmental samples which consist mostly of microbial dark matter [12]. Efforts to squeeze out subspecies resolution from 16S rRNA gene sequencing data mostly relate to improvements in bioinformatic pipelines enabling predictions based on single-nucleotide sequence variants [13, 14].

Meaningful comparative analysis of microbial communities requires accurate quantification of taxa for reliable numerical comparison across samples. Different strategies have been introduced to determine relative abundances of identified taxa in complex samples. It was demonstrated that 16S rRNA gene sequences and other targeted approaches perform poorly for such purposes because of inherent biases [15]. In contrast, the numbers of reads in metagenomic data might be normalized by genome size [16], if taxonomically annotated reference genomes are available, and provide accurate abundance estimates. Alternatively, read coverage of the selected single-copy clade-specific genes might be used to infer quantitative abundances without further normalization [17]. Such methods are accurate but require extensive prior knowledge of genome sequences and thus are considered as not compatible with poorly characterized taxa, although improvements in computational algorithms were recently suggested to overcome this limitation [18].

We hypothesized that linking highly conserved 16S rRNA gene sequences with regions of lower conservation might overcome limitations related to both targeted 16S rRNA analysis and metagenomic sequencing. Specifically, we sought to improve taxonomic resolution and abundance estimation retaining the cost-effectiveness of targeted approaches. Here, we describe a new method of high-throughput microbial profiling which captures 16S rRNA hypervariable regions along with the genomic sequences upstream of the gene. We termed this technique semi-targeted 16S sequencing, or st16S-seq. We first performed a global *in silico* analysis of regions upstream the 16S rRNA gene and confirmed their diagnostic potential. Next, we validated the proposed approach on microbial community DNA standards. Finally, we challenged st16S-seq with ultra-high-complexity environmental samples and benchmarked our approach against the established amplicon sequencing and whole metagenome sequencing techniques.

## Methods

### Dataset for *in silico* analysis

#### Dataset preparation

The dataset was prepared from genome sequences located in the NCBI Genome database [19]. First, metadata for bacterial genome assemblies were downloaded. Next, for each unique strain only the best assemblies were chosen, discarding incomplete genomes and prioritizing newly released assemblies over older ones as well as reference genomes over representative and unannotated ones. Incomplete genomes were discarded due to the potential lack of 16S rRNA gene sequences or incomplete gene copy representation. Once the assemblies were filtered, FASTA sequences together with their corresponding GCC annotations were downloaded. Using this strategy 15 669 genomes, each representing a unique strain, were obtained.

16S rRNA and upstream sequences were extracted from genomes based on GCC annotations. Coordinates for the 16S rRNA gene were searched in the GCC file by matching a ‘16S ribosomal RNA.*’ string. Next, genes were filtered based on their length; only genes of length 1000–2000 bp were used. All retrieved sequences were automatically oriented in the 5′−3′ direction with the beginning of the gene located in the 5′ terminus of the sequence.

From genome assemblies 16S rRNA genes were retrieved for 15 655 strains. Four assemblies had missing/empty files and 52 16S rRNA gene sequences were too short or too long. Two generated sequence files (one for 16S rRNA and another for upstream region sequences linked to their corresponding 16S rRNA genes) were matched – this was done to check for partial upstream sequences. No sequences were removed during this step. In total, 80,014 16S rRNA gene sequences linked to their upstream genomic regions were extracted from the genomes of 15 655 strains.

#### Extraction of 16S rRNA hypervariable regions

16S rRNA sequences were split into variable sub-regions confined by specific primers. Primer sequences were chosen based on previously published data [20]. For each conservative region, except the one between V1 and V2 sequences, primers were selected based on their coverage across species (Table S4). Primer locations were searched across 16S rRNA sequences using the blast algorithm. For each variable region within each 16S rRNA gene sequence, a single primer with the highest bitscore was chosen. Primers with more than two mismatches were discarded. For each sequence, primer alignment locations were saved, and these locations were later used to split sequences into corresponding hypervariable regions. Primers were matched to the positions between hypervariable regions. Each primer was named after the region located upstream of the primer hybridization site.

Most sequences were lacking the primer intended to amplify the V1 sequence (424 sequences) (Table S5). A substantial part of these mismatches were 3 nt in size – 1 nt above the cutoff. Some sequences were missing all nine primers, and such sequences were unlikely to be 16S rRNAs and probably occurred due to incorrect GCC annotations.

For quality control of detected primer positions, the locations of all primers were checked to be in sequential order (e.g. V10 is after V9, V9 is after V8). The locations of two primers for two sequences were found to be non-sequential.

Finally, whole genomes were removed if at least a single 16S rRNA sequence belonging to the genome was discarded for one of the previously stated reasons. After removing sequences (and genomes) with missing primers and non-sequential primer locations, 15 376 strains and 78 925 sequences remained in the dataset.

#### Taxonomy matching

To match taxonomic lineage to each strain, NCBI taxonomy IDs supplied with genome assemblies were matched to the NCBI taxonomy database. Retrieved taxonomic information was used to filter genome assemblies based on the following criteria: (i) information about taxonomic lineage was not present in the taxonomic database; (ii) and the assembly did not have a specified genus or species. After taxonomy matching and filtering, 13 595 unique genome assemblies and 71 254 16S rRNA sequences remained in the dataset.

#### Removal of ambiguous sequences

16S rRNA sequences containing any wildcard letters were discarded. This was done to avoid ambiguous sequence representations.

In total, 17 555 assemblies were analysed as potential members of the dataset. Only full genomes representing unique strains were downloaded and processed further. Of 15 669 initial genomes, 2129 were discarded during several filtering steps. Stepwise changes in sequence and genome counts within the built dataset are depicted on Fig. S1.

### *In silico* analysis of genomic sequences upstream of 16S rRNA gene

#### Conservation profile of 16S rRNA gene sequences and upstream regions

Positional variability of dataset sequences was analysed by calculating Shannon entropy for multiple sequence alignment (MSA) columns. First, all sequences in the database were aligned by Clustal Omega v1.2.2 [21], and then columns of MSA were removed or retained based on reference sequence. For retained columns Shannon entropy was calculated as: $S=-\sum_{i}P_{i}log_{4}P_{i}$

Here, *P*_*i*_ is a fraction of times nucleotide *i* appears in a column of MSA; log with base 4 is used to normalize values to 1. Shannon entropy value of 1 indicates that all nucleotides appear in a column an equal number of times (in this case 25 %). An *S* value of 0 indicates that only a single nucleotide appears in an MSA column (100%).

#### Analysis of identifiable 16S rRNA gene copy numbers

The following dataset normalizations were used for the calculation of identifiable gene copies: (i) intra-strain, inter-strain and inter-species: the number of strains was limited to 10 per species; (ii) inter-strain and inter-species: only species with at least two strains were used; (iii) inter-species: the number of species was limited to 10 per genus. Normalizations limiting the number of species per genus or strains per species to 10 were applied to 13 % of genera and 6 % of species within the dataset. To accurately account for 16S rRNA gene copy number variation, each gene copy should produce a unique contig with at least a single nucleotide sequence variant. Computational assessment of identifiable 16S rRNA copies at different taxonomic levels was performed by dividing a number of unique sequence variants within each member of the selected rank by the number of total sequence variants, and the mean of all members was taken. We considered this as a fraction of identifiable gene copies. For example, if strain X has a single 16S rRNA gene copy, for strain X 100 % of copies will be identifiable. Meanwhile, if strain Y has two identical gene copies, 50 % of sequences will be identifiable. If strains X and Y make up the whole dataset, then for this dataset 75 % of sequences will be identifiable.

#### Clustering into OTUs

The quality of clustering sequences into OTUs was assessed by published methods [5]. Briefly, four metrics were used: (i) richness ratio – a ratio between generated OTUs and the number of unique species, defined as min⁡(S,O)max⁡(S,O) where *S* corresponds to the number of species and *O* to the number of OTUs; (ii) Matthews’ correlation coefficient defined as the correlation between real and predicted values identified by the classifier; (iii) bijection – the fraction of species that have all their sequences assigned to the same OTU with no other false sequences present; and (iv) normalized mutual information – a measurement of mutual dependence between frequency distribution pairs. To calculate clustering metrics, we first split sequences into subsequences representing regions of interest. Then, subsequences were aligned by Clustal Omega. Clustering into OTUs was performed at various clustering percentages by Mothur v1.43.0 [22]. For each metric the optimal clustering percentage was calculated. Clustering was performed with two sets of sequences: (i) all sequences of our database and (ii) representative sequences where each species is represented by a single randomly selected strain.

#### Sequence classification accuracy

To assess classification accuracy based on various combinations of 16S rRNA and near-16S sequences, the Ribosome Database Project (RDP) classifier [23] with 80 % bootstrap cutoff was used, employing a leave-clade-out testing approach. All sequences of the database built in this study were used for classification. The classifier was retrained on the sequences corresponding to each specific region under investigation. Fivefold cross-validation was performed changing the training and validation dataset compositions for each iteration. Classification accuracy was defined as the ratio of accurate predictions (>80 % bootstrap) to all predictions.

#### Primer design

Primer sequences were designed using an in-house pipeline based on silva release 132 [24] dataset ‘SSU Ref NR 99’. The sequences of the dataset were clustered at the 95 % identity threshold with vsearch v2.15.1 [25]. The sequences containing non-standard symbols were discarded from the dataset supplied for the design algorithm. The algorithm selected primer sequences in a way which maximizes sensitivity ensuring that the annealing temperature would not drop below a certain threshold due to mismatches. All primers were synthesized by Metabion GmbH; the full sequences are provided in Table S6.

#### Synthesis of oligonucleotide-tethered dideoxynucleotides

All reaction components were added to the reaction mixture as solutions in water unless specified otherwise. Modified oligonucleotide of the sequence 5′-hexynyl-AGATCGGAAGAGCACACGTCTG-biotin-3′ (ON) was synthesized by Metabion GmbH requesting HPLC purification.

A solution of 5-(3-(2-azidoacetamido)prop-1-ynyl)−2′,3′-dideoxycytidine-5′-triphosphate or 5-(3-(2-azidoacetamido)prop-1-ynyl)−2′,3′-dideoxyuridine-5′-triphosphate (3 eq.) solution was added to ON (200–210 nmol) solution in sodium phosphate buffer (1 ml, 100 mM, pH 7). A premixed solution of CuSO_4_ (100 mM, 12 eq.) and THPTA (250 mM, 5 eq. to CuSO_4_) was then added to the reaction mixture, followed by the addition of sodium ascorbate (1 M, 50 eq. to CuSO_4_). The reaction mixture was stirred for 20 min at 42 °C, and quenched with 0.5 M EDTA-Na_2_ solution (1 ml, pH 8). The products were purified by C18 reversed-phase chromatography using 100 mM TEAAc/ACN (11–18 %) as eluent and desalted using water/ACN (0–100 %) as eluent.

The oligo-modified ddC^ON^TP was obtained with 39 % (82 nmol) yield. HRMS (ESI^-^): calculated monoisotopic mass for [M]: 7916.345; found: 7916.342. The oligo-modified ddU^ON^TP product was obtained with 34 % (67 nmol) yield. HRMS (ESI^-^): calculated monoisotopic mass for [M]: 7917.329; found: 7917.326.

The synthesis principle and structure of oligonucleotide-tethered dideoxynucleotides is depicted in Fig. S4. Oligonucleotide-tethered dideoxynucleotides used in this study are available upon request from the authors.

### st16S-seq library preparation

#### Samples

Two types of samples were used in this study: (i) well-characterized microbial community DNA standards: 20 Strain Even Mix Genomic Material (ATCC MSA-1002) and ZymoBIOMICS Microbial Community DNA Standard (Zymo Research, D6305); and (ii) Cambisol soil samples collected in grasslands (Soil #1, Soil #4 and Soil #5), near the forest (Soil #2, Soil #6) and in a cultivated field (Soil #3). Soil samples were collected from ~30 cm soil depth in early August and stored frozen until use. DNA from 250 mg of soil was extracted using ZymoBIOMICS DNA Miniprep Kit (Zymo Research) according to the manufacturer’s recommendations. DNA quality and quantity were assessed by using a NanoDrop 2000 spectrophotometer (Thermo Scientific). No template control (NTC) samples, with DNA replaced by nuclease-free water, were included in all experiments to account for background amplification.

#### Primer extension

Each library was generated with 30 ng of sample DNA. DNA was mixed with 10 pmol of equimolar primer mix described above in a 20 µl reaction mixture containing 40 U Thermo Sequenase enzyme with thermostable inorganic pyrophosphatase (Thermo Scientific), 4 µl Thermo Sequenase Reaction Buffer (Thermo Scientific), 500 pmol dNTP mix (Thermo Scientific), 5 pmol ddU^ON^TP and 2 pmol ddC^ON^TP. The reaction was incubated in a thermocycler with the following temperature conditions: 95 °C for 4 min, followed by 15 cycles of linear extension at 95 °C for 1 min, 65 °C for 30 s, 72 °C for 1 min and final extension at 72 °C for 5 min. Making use of the 3′ biotin modification within the oligonucleotide-tethered dideoxynucleotide, primer extension products were purified by affinity capture using Dynabeads M-270 Streptavidin beads (Thermo Scientific) according to the manufacturer’s instructions for immobilization of nucleic acids. Elution was performed for 5 min at 95 °C in 20 µl nuclease-free water.

#### Amplification

As primer extension products are labelled by partial adapter sequences at both termini, they are compatible with a standard indexing PCR. In total, 19 µl of streptavidin-purified DNA was mixed with 25 µl of Invitrogen Collibri Library Amplification Master Mix (Thermo Scientific), 20 U of 3′−5′ exonuclease-deficient Phusion enzyme (1 µl) and 5 µl of indexing primers (50 pmol each) of the following sequences: i5 primer: 5′-AATGATACGGCGACCACCGAGATCTACACTCTTTCCCTACACGACGCTCTTCCGATCT-3′; i7 primer: 5′-CAAGCAGAAGACGGCATACGAGAT[8nt_index]GTGACTGGAGTTCAGACGTGTGCTCTTCCGATCT-3′.

Phusion exo- was added as a helper enzyme to ensure efficient synthesis through the unnatural linker within the oligonucleotide-tethered dideoxynucleotide (related data will be reported elsewhere). Cycling was performed as follows: denaturation at 98 °C for 30 s, followed by 20 cycles of denaturation at 98 °C for 10 s, annealing at 60 °C for 30 s, extension at 72 °C for 1 min and final extension at 72 °C for 1 min. Each PCR was then purified using Dynabeads Cleanup Beads (Thermo Scientific). DNA binding to the beads was performed by mixing 45 µl of bead suspension with 50 µl of sample and subsequent incubation at room temperature for 5 min. Each sample was then placed on a magnet, supernatant was removed and beads were resuspended in 50 µl of elution buffer containing 10 mM Tris-HCl (pH 8). Then, 50 µl of fresh beads was added again to the sample and binding was repeated. After incubation at room temperature, each sample was placed on a magnet, supernatant was removed and beads were washed twice with 85 % ethanol. To elute libraries, beads were resuspended in 22 µl of elution buffer and incubated for 1 min at room temperature. Then, 20 µl of the supernatant was transferred to the second amplification step.

The second amplification step is required to generate enough material for sequencing. Each sample was amplified in a 50 µl reaction with Invitrogen Collibri Library Amplification Master Mix with Primer Mix (Thermo Scientific) for 12 cycles according to the recommended temperature conditions. Final libraries were purified using Dynabeads Cleanup Beads (Thermo Scientific). DNA binding conditions were changed so that 30 µl of bead suspension was used in the first binding step, and 50 µl of bead suspension in the second binding step. Fragment size distribution was then assessed via the Agilent Fragment Analyzer system (Agilent Technologies) with an HS NGS Fragment kit. Quantification of sequenceable molecules was performed with an Invitrogen Collibri Library Quantification Kit (Thermo Scientific).

#### WGS library preparation

For comprehensive characterization of soil samples, whole metagenome libraries were prepared from 10 ng of soil DNA. DNA was sheared to ~300 bp using the Covaris E220 Focused-ultrasonicator (Covaris) and processed through the Invitrogen Collibri PS DNA Library Prep Kit for Illumina Systems (Thermo Scientific) workflow. Fragment size distribution was then assessed by the Agilent Fragment Analyzer system (Agilent Technologies) with HS NGS Fragment kit. Quantification of sequenceable molecules was performed with an Invitrogen Collibri Library Quantification Kit (Thermo Scientific).

### 16S rRNA gene amplicon library preparation

To compare the semi-targeted approach with conventional amplicon sequencing of one or several 16S rRNA gene hypervariable regions, ZymoBIOMICS Microbial Community DNA Standard, ATCC MSA-1002 DNA and soil DNA were used for library preparation with commercially available microbiome profiling kits or publicly available protocols (Table S7). All procedures were executed with strict adherence to the manufacturers’ instructions.

### Sequencing

The 2×300 bp paired-end (PE) sequencing was performed on the Illumina MiSeq instrument using MiSeq Reagent Kit v3 (600-cycle) for semi-targeted and 16S rRNA gene amplicon libraries. Whole genome sequencing (WGS) libraries were sequenced with a MiSeq Reagent Kit v2 (300-cycle) at 2×150 bp PE mode.

### Processing of st16S-seq reads

An overview of the st16S-seq data analysis pipeline is depicted in Fig. S9.

#### Read preprocessing

1. Read quality trimming and sequencing adapter removal steps were executed using the BBDuk program from BBmap v38.87 [26]. For further analysis only reads that were longer than 250 nt were used. The command line options for BBDuk excluding file inputs/outputs were as follows ‘ktrim=r k=23 mink=11 hdist=1 minlength=250 maxns=0 qtrim=r tpe tbo’. For soil samples, the quality limit ‘trimq’ was set to 15, while for mock community samples this was set to 20.

2. The reads that passed initial preprocessing were subjected to further filtering steps. (a) First, read pairs that do not contain expected 16S rRNA targeting reads were discarded (BBDuk options ‘k=15’). (b) The remaining reads were subjected to the detection of an expected 16S rRNA region using the nhmmer program from the HMMER v3.3 package [27]. The nhmmer search was conducted using a profile hidden Markov model that was constructed based on silva release 132 [22]. The model was created using the sequences matching silva’s ‘SSU Ref NR 99’ dataset clustered to the 95 % identity threshold with vsearch v2.15.1 [23]. (c) The reverse reads (R2) were checked for the presence of 16S rRNA primer sequences and discarded if found at the beginning of the read (BBDuk option: ‘hammingdistance=1 restrictleft=40’). (d) The sequences of the 16S rRNA targeting primers were removed from the forward reads (R1), restricting the search of matches to the beginning of the reads and using permissive detection (BBDuk options: ‘ktrim=l k=12 restrictleft=50 mink=7 edist=2’). (e) The forward and reverse reads after independent filtering steps were rematched using fastq_pair v1.0 [28].

3. Post-filtering manipulations. (a) Reads were subjected to an error correction step using SPAdes v3.13.1 [29]. (b) Overlapping reads were joined using BBMerge from the BBmap v38.87 package [30]. If a read pair was joined using BBMerge, the resulting sequence was considered as a forward read while the matching reverse read was generated as its reverse complement using seqkit v0.8.1.

Reads were then analysed in two ways either targeting assembly of near-16S regions or quantification. These are briefly described below.

1. Quantification per phylogenetic group was based only on the reads that had an overlapping 3′ terminus and were joined. Identical sequences were removed using vsearch v2.15.1 and further grouped by swarm v.3.0.0 [31, 32] (non-default parameter ‘d=1’). Taxonomic assignment using DADA2 v.1.14 [33] was based on the first 240 bp of the reads after the two clustering steps.

2. Assembly of near-16S regions. (a) All reads after the error correction and joining steps were clustered using vsearch and swarm v.3.0.0 tandem (as above) based on the first 240 bp from the forward reads. In this case swarm was run allowing larger differences (‘d=2’). (b) The reads were grouped into sets (OTUs) matching the two-step clustering. Read names after error correction and overlapping 3′ end joining were mapped to the clusters after consecutive vsearch and swarm processing using a julia v1.5 script. The script integrated the two USEARCH cluster format files produced by vsearch and swarm. Only those clusters comprising no less than 0.001 of read pairs after filtering steps were subjected to assembly. Before the assembly, the reads were quality trimmed using a more restrictive cut-off with BBDuk (command line option ‘trimq=20’). (c) The assembly itself was conducted exploiting programs from the BBmap v38.87 package.

i. Reads were merged using BBMerge, utilizing the possibility to perform local assemblies and merge non-overlapping paired-end reads. The assemblies/mergers were done under several *k* values (150, 190, 220). Other options used with BBMerge were as follows: ‘mincountseed=1 mincountextend=1 rem ecct iterations=20 vstrict=t’. After assembly, contigs shorter than the 75th length percentile were discarded. All contigs produced using different *k* values were collected for further processing.

ii. If BBMerge failed to assemble any contigs, it was attempted to obtain contigs using only forward reads by extending them using the Tadpole program (command line options: ‘tmincountseed=1 mincountextend=1 mode=extend er=1000’). The extensions were conducted using varying *k* values (70, 150, 190, 220).

iii. The contigs resulting from the two initial steps were clustered using vsearch with the ‘derep_fulllength’ command.

iv. The dereplicated reads were subjected to an additional extension step with sequences from reverse reads from non-overlapping pairs using the Tadpole program (command line options: ‘tmincountseed=1 mincountextend=1 mode=extend er=1000’). The extensions were conducted using varying *k* values (70, 150, 190, 220). All contigs produced using different *k* values were collected for further processing.

v. The contigs were then clustered by vsearch using the ‘cluster_fast’ command at a 0.97 identity level.

vi. The resulting contigs were subjected to an error correction step by Tadpole using reverse reads from the non-overlapping read pairs (non-default command line options ‘aggressive=t k=20’).

vii. Finally, the reads were again clustered by vsearch using the ‘cluster_fast’ command at a 0.97 identity level and the corresponding centroids were considered as the final contigs for further analysis.

3. The assembled contigs were then further subjected to several cleanup steps in order to remove artefactual contigs. Cleanup steps were performed at two levels: within the contigs resulting from one read cluster based on 240 bp Step (2a) and then considering contigs from all clusters as one set.

(a) Cleanup of contigs resulting from one read cluster:

i. The contigs originating from each initial clustering were clustered based on the first 230 bp using consecutive clusterings with swarm (‘d=2’) and vsearch (‘cluster_fast’ command, identity cutoff 96 %). Only the contigs that were included in this cluster were used for further analysis. In this way, all contigs that had an atypical/artefactual 5′ starting sequence were discarded.

ii. Contigs that were shorter than 52 % of the maximum contig length were discarded.

iii. Contigs that are ‘contained’ within other contigs were discarded. A contig was considered to be overlapping if the identity level of its fragment excluding the last 20 bp was more than 98 % and the aligned fraction constituted ≥ 0.9 of the whole contig’s length.

(b) Cleanup of contigs from all clusters as one set:

Contigs were aligned using the blastn program from the blast v.2.10.1 package against the whole nt database (downloaded October 2020). Options were default except the set limitation to a maximum of three target sequences. The contigs were considered to be correct if the following conditions were met:

1. The aligned fraction constituted ≥ 90 % of the contig length.

2. The match against a database sequence started from at most the fifth base from the 5′ terminus.

3. The matched sequence in the database in the matched region contains fewer than 5 ‘Ns’ in the sequence.

4. Each contig was assigned a genus based on the best matching sequence in the database. The genus was determined based on the taxon ID of the matching sequence using TaxonKit [34]. Within a contig group originating from the same read cluster (2a) for each detected genus the median of the corresponding contig’s length was calculated. The genus with the largest median length of the contigs assigned to it was considered to be the typical one, and the contigs that matched other than the typical genus were discarded. Moreover, at this stage contig 3′ ends that span beyond the matched region in the blast search were trimmed off.

5. The remaining sequences were again checked for consignments discarding the contigs that are ‘contained’ by other sequences at the same time trying to keep sequences that start to differ at the very end of the 3′ terminus potentially indicating different 16S rRNA gene copies. A contig was discarded: (a) if identity was ≥ 96 % across 99 % of its length compared to a longer contig; (b) if contigs were of the same length, the one with higher abundance was kept. Abundance (in terms of TPM - transcripts per million) was evaluated using salmon v1.3.0 [35] with the non-default parameters: ‘--posBias --biasSpeedSamp 1 --forgettingFactor 1.0 --useEM’ and using alignments produced by bowtie2 v2.4.1 [36] (non-default options: ‘-X 2000 --very-fast’. Only alignments matching proper pairs and with MAPQ ≥ 1 were considered. Alignment filtering was conducted using samtools v1.10 [37] (command line options ‘-f 2 -q 1’). The alignments were done mapping all reads that contained the expected 16S rRNA fragment (2e) against all assembled contigs (2c). (c) Identity at the 3′-terminal fragments (15 and 50 bp in length) in the alignment was ≥ 80 %. This enabled keeping long contigs that started to differentiate among different 16S rRNA copies only at around 0.8–1 kb (exclusing the genera *Enterococcus* and *Pseudomonas *) from the primer binding sites.

(c) Relative abundance of the clusters (OTUs) resulting from the swarm-based clustering of the first 240 bp of the forward read (2a) (denoted as *a*_f_) was evaluated as follows:

$a_{f}=\frac{\sum_{i=1}^{j}\frac{n\left(b_{j}⋂a\right)}{n\left(b_{j}\right)}}{j}$ , where *b* is names of reads of a cluster (described in section 1) after deduplication with swarm of merged read pairs; *a* is names of reads of a cluster after clustering based on the first 240 bp of the forward read with swarm (described in section 2a); *j* is the number of *b* clusters; and *n* is the number of read names.

### Analysis of data produced by conventional 16S rRNA sequencing methods

1. Read quality trimming and sequencing adapter removal steps were performed using the BBDuk program from BBmap v38.87 [24]. Only those reads that were longer than 50 bp were used. The command line options for BBDuk excluding file inputs/outputs were as follows: ‘ktrim=r k=23 mink=11 hdist=1 minlength=50 maxns=1 qtrim=r trimq=15 tpe tbo’.

2. Overlapping reads were joined using BBMerge from the BBmap v38.87 package [28].

3. The merged reads were clustered into OTUs using swarm v.3.0.0 [29, 30] (non-default parameter ‘d=2’) after de-replication with vsearch v2.15.1 with ‘derep_fulllength’ command.

4. Clusters containing fewer than two sequences were discarded using the vsearch ‘sortbysize’ command.

5. Chimeric sequences were discarded by consecutive use of vsearch ‘uchime_denovo’ followed by reference-based chimera removal using vsearch ‘uchime_ref’ and reference sequences from the ‘Gold’ database.

The data produced with QIAseq 16S/ITS Screening Panel and Swift Amplicon 16S+ITS Panel kits were processed following the manufacturer’s guidance as those kits generate more than one amplicon.

### Phylogenetic assignments

Phylogenetic assignments of the clustered reads were performed in two ways.

**Fig. 1.**
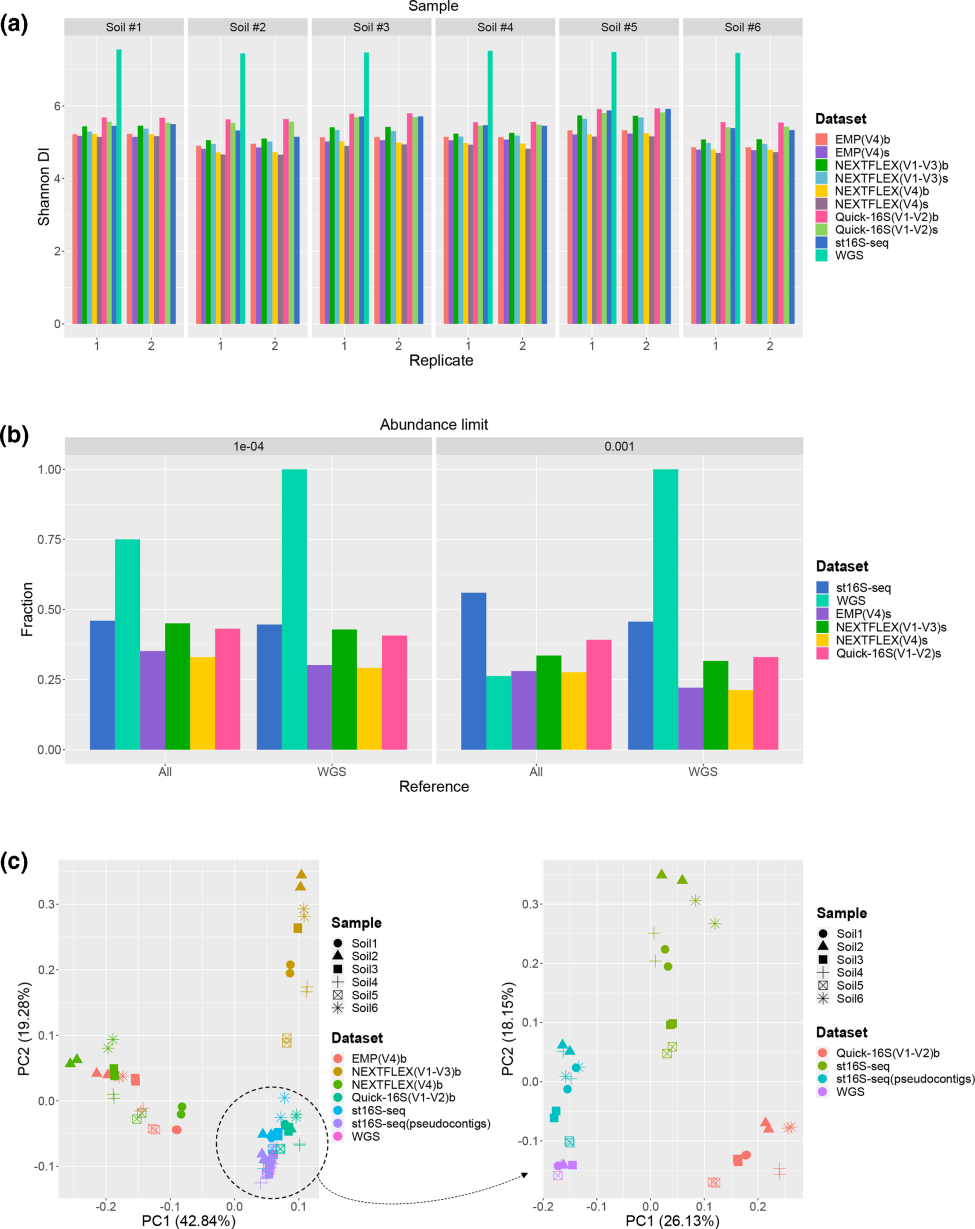
Species-level discriminatory power of st16S-seq on complex samples and comparison with conventional techniques. (a) Shannon diversity indices obtained for six soil samples sequenced employing various library preparation approaches. (**b**) The fractions of taxa detected in each dataset as compared to either the total number of taxa identified in all soil samples by all sequencing methods (reference value ‘All’) or to taxa identified only by whole metagenome sequencing (reference value ‘WGS’). The analysis was conducted considering taxa for which abundance was above the defined minimum thresholds. (**c**) Principal components analysis considering the relative abundance of reads assigned per bacterial species across different soil samples processed by various library preparation techniques. The graph on the right depicts data that cluster near WGS. The dataset label ‘b’ stands for the downsampling level equivalent to the amount of on-target reads in st16S-seq datasets. The same downsampling strategy is true for WGS samples in all cases. The dataset label ‘s’ denotes the downsampling level equivalent to the number of unique reads in st16S-seq datasets retained after deduplication. The data for st16S-seq correspond to unique on-target reads in all cases.

**Fig. 2.**
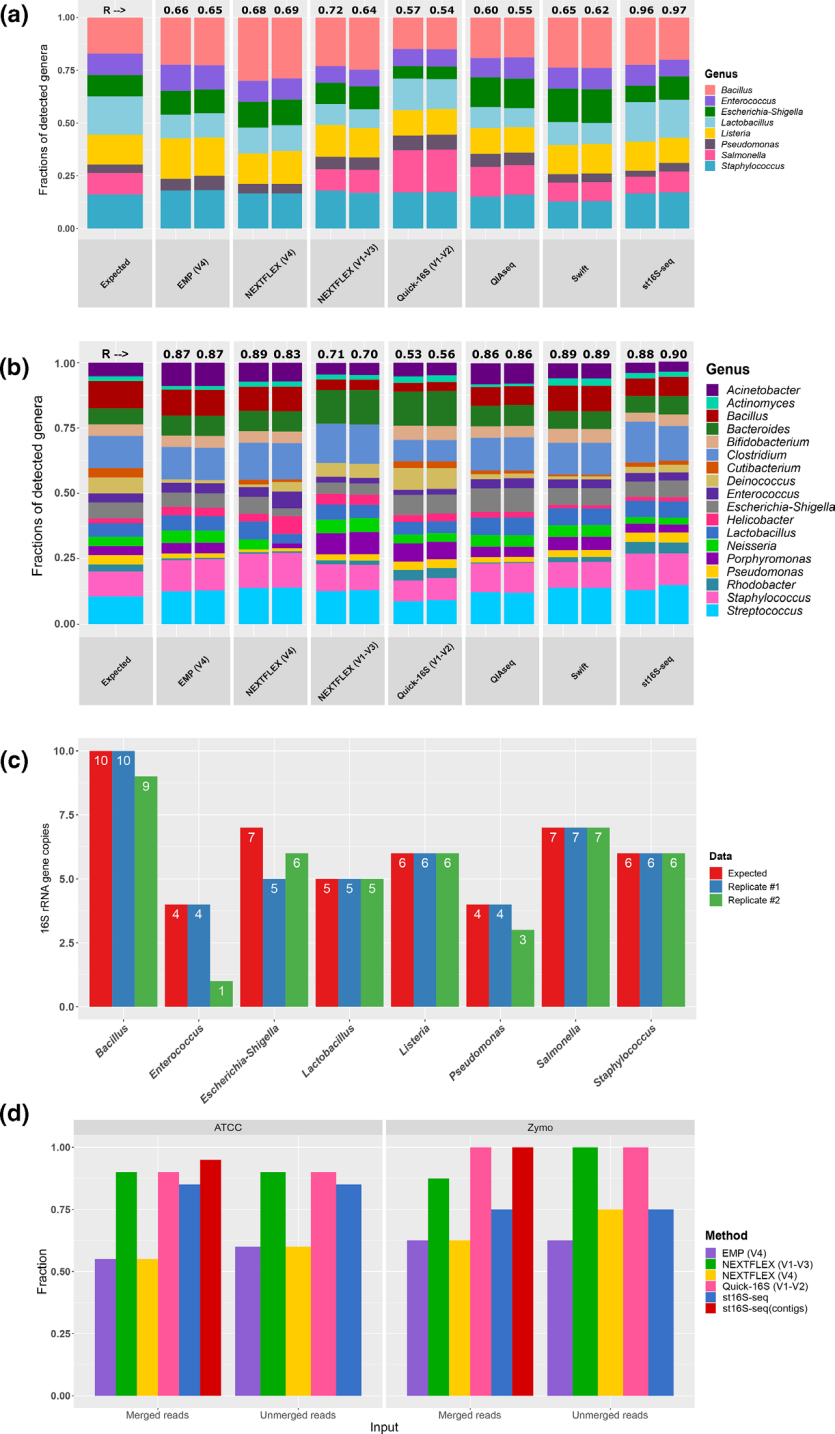
Validation of st16S-seq on mock community DNA standards and comparison with conventional techniques. (a) Read distribution across bacterial genera in libraries prepared from ZymoBIOMICS Microbial Community DNA standards with various commercially available kits and st16S-seq approach. Numbers above bars indicate Pearson’s correlation coefficients between the expected and obtained read distributions. Two replicates are shown for each sample. (**b**) Read distribution across bacterial genera in libraries prepared from ATCC microbiome standard (ATCC MSA-1002) DNA with various commercially available kits and the st16S-seq approach. Numbers above bars indicate Pearson’s correlation coefficients between the expected and obtained read distributions. Two replicates are shown for each sample. (**c**) The number of 16S rRNA gene copies detected by st16S-seq within genomes of the members of the ZymoBIOMICS Microbial Community DNA standard that equates to the number of 16S rRNA contigs after removal of artefactual sequences. (**d**) Species-level characterization of mock microbial communities using the NCBI database as a reference and either unmerged reads or only merged reads as an input for Kraken. Bars represent fractions of identified species as compared to the expected compositions.

1. In order to compare whole metagenome sequencing data with the semi-targeted data of st16S-seq and targeted sequencing data of other 16S rRNA kits, Kraken 2.0.7 [38] was used. It was recently demonstrated that Kraken2 is well suited not only for WGS, but also for targeted sequencing data [39]. To compare the ability to reveal the composition of complex samples (Fig. 1) based on data originating from different sample preparation methods, the Kraken classification of reads was conducted in the same manner using the same reference database. The subset of bacterial sequences of the standard Kraken database was used as a reference (downloaded in November 2020). After the Kraken runs where the input was read pairs, quantities at the species and genus levels were estimated by Bracken 2.6 [16]. In addition to standard Kraken2 use, the st16S-seq data were supplied for classification in two additional ways: (i) concatenated set of cleaned up contigs, and (ii) concatenated reads from pairs that were used for 16S rRNA contig assemblies. The concatenation of sequences was conducted using the fuse.sh program from the BBmap v38.87 package [28].

2. To generate data for mock microbial communities (Fig. 2), taxonomic assignment was conducted using DADA2 v.1.14 [31]. The silva 138 database was used as a reference. The non-standard options were ‘inBoot=40 tryRC=TRUE’.

### Note on downsampling

Analysis of the soil samples was conducted after downsampling to 130 000 read pairs after a quality trimming step. In addition, the reads were downsampled to the median number of reads in st16S-seq samples after read processing/filtering (as indicated in the main text). During analysis of the ZymoBIOMICS and ATCC mock communities, the 280 000 read pairs were used after quality trimming steps. Downsampling was executed using the ‘subseq’ program of seqkit v0.8.1.

## Results

### Adjacent genomic sequences enhance discriminatory power and allow accurate quantification when combined with 16S rRNA hypervariable regions

To assess whether capturing additional genomic sequences along the 16S rRNA gene is a beneficial microbiome profiling strategy, we *in silico* extracted 80 014 16S rRNA sequences and corresponding 1 kb upstream regions from 15 655 publicly available bacterial genome assemblies. After several filtering steps (Fig. S1, available in the online version of this article, see Methods), we built a database of taxonomically annotated sequences consisting of 71 035 16S rRNA and near-16S regions from 13 570 unique genome assemblies. The distribution of strains within the final dataset was skewed towards well-characterized species: ~50% of strains represented only 31 species with most strains belonging to *Escherichia coli* and *Salmonella enterica *. The number of strains per species was therefore limited to 10 for certain analyses, such as the assessment of identifiable 16S rRNA gene copy numbers, to avoid potential biases.

**Fig. 3.**
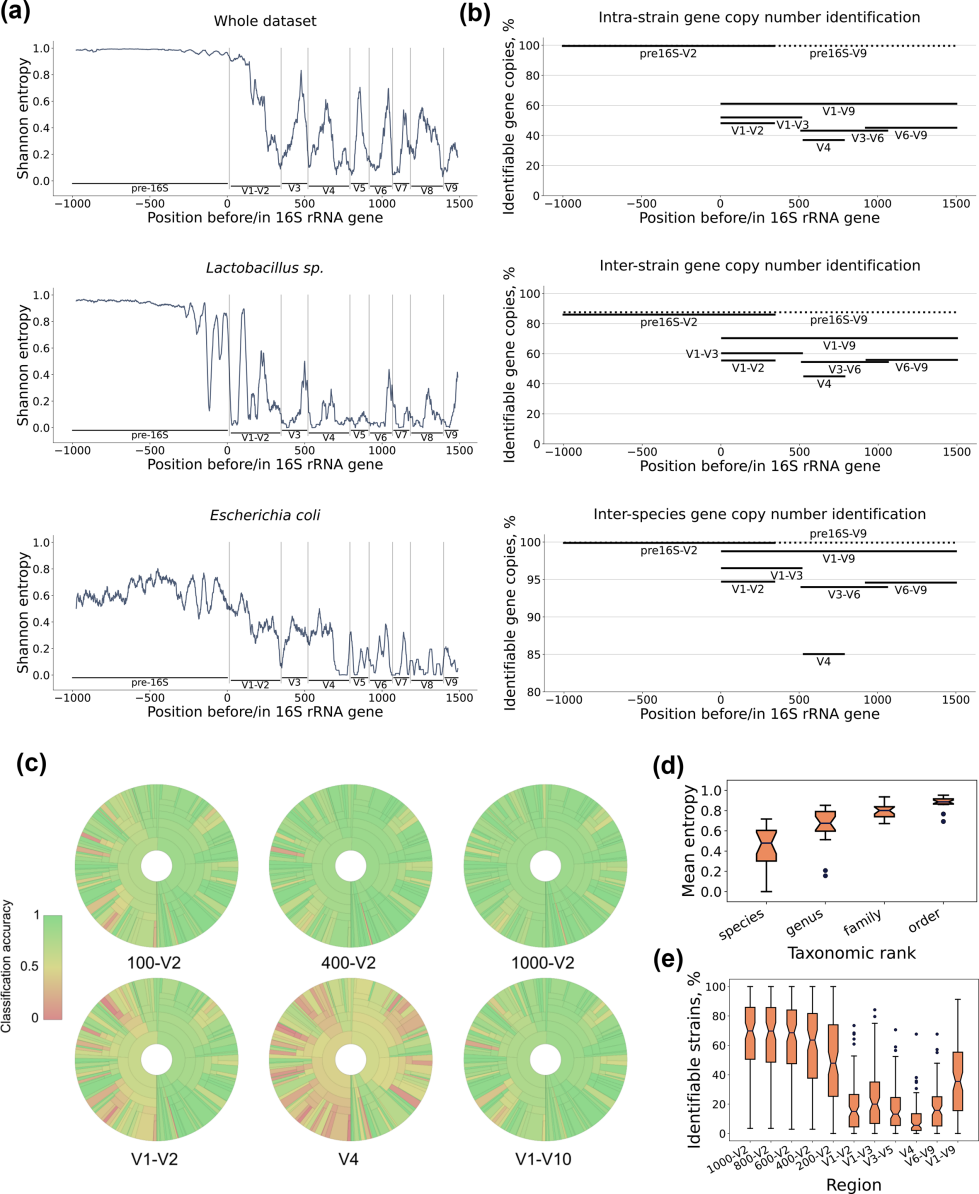
Discriminatory power of genomic regions upstream of the 16S rRNA gene. (a) Shannon entropy values of sequence regions upstream and within the 16S rRNA gene. Multiple sequence alignments were built on the basis of the database created in this study. (**b**) The percentage of identifiable 16S rRNA gene copy numbers as assessed by various regions of the 16S rRNA gene. (**c**) Krona charts depicting *in silico* estimated classification accuracy at the species level. The outer ring corresponds to the genus/family level. The size of circular fragments is proportional to the number of sequences belonging to the rank. For near-16S sequences, the length of included genomic fragments is indicated (100, 400, 1000 bp). In all cases, near-16S regions were linked to V1–V2 16S rRNA sequences. (**d**) Distribution of mean Shannon entropy values at different taxonomic ranks as assessed for sequences upstream of the 16S rRNA gene. Each boxplot represents 20 members of the taxonomic rank, with maximum number of five sub-members for each member of the rank and up to two strains per species. Centre line – median, box limits – upper and lower quartiles, whiskers – 1.5× interquartile range, points – outliers. (**e**) Distribution of fractions of identifiable strains within species. Each boxplot represents 100 species with the highest number of strains. For near-16S regions, the upstream fragment length (1000, 800, 600, 400 and 200 bp) is indicated. Near-16S regions in all cases were linked to the V1–V2 16S rRNA sequences. Centre line – median, box limits – upper and lower quartiles, whiskers – 1.5× interquartile range, points – outliers.

The analysis of positional sequence variability revealed that genomic sequences adjacent to the 16S rRNA gene are generally less conserved than within-gene sequences, but for single species the conservation level of near-16S regions is higher (Fig. 3a). Moreover, the entropy varies between different members of the rank. Despite such variability, the average entropy of near-16S genomic regions increases significantly for higher taxonomic ranks with *P* < 0.05 for all of species vs. genus, genus vs. order and order vs. family differences as assessed by one-sided Mann–Whitney U test. This reflects the decrease of phylogenetic relatedness between genome sequences of higher taxonomic ranks (Fig. 3d).

We next assessed the differentiation of 16S rRNA gene copies on intra-strain, inter-strain and inter-species levels. Within individual genomes, 16S rRNA hypervariable regions were able to differentiate between up to ~50% of gene copies. The full-length gene sequence raised the fraction of identifiable copies to only 60%. The inclusion of near-16S regions in conjunction with either V1–V2 or V1–V9 increased the discrimination rate to 99.7%. Microbiome samples often contain a mixture of closely related bacterial lineages, which complicates quantitative assessment because of the presence of identical 16S rRNA genes in genomes of different strains. To evaluate inter-strain 16S rRNA gene copy differentiation, we included species with at least two strains in the analysis in order not to inflate the mean with single strain variants. Although neither sequence allowed for absolute discrimination, we observed > 80 % of identifiable 16S rRNA copies for the near-16S and 16S rRNA sequence combination in contrast to ≤ 70% for within-gene sequences. At higher taxonomic level, inter-species sequence variation somewhat lowers the discriminatory power of full-length 16S rRNA gene sequences and sequences of hypervariable regions, although there is almost no impact on near-16S regions, thus allowing for absolute gene copy number identification (Fig. 3b). The discriminatory power of near-16S regions depends on the fragment length included in the analysis. We evaluated up to 1 kb upstream of the 16S rRNA gene in conjunction with 341 bp within the gene corresponding to V1 and V2 sequences. We observed that copy number identifiability peaks at 559 bp upstream of the 16S rRNA gene and does not significantly increase with a further increase in fragment length (Fig. S2). The same trend was observed for the discrimination between strains.

The ability of near-16S along with 16S rRNA sequences to distinguish between strains was assessed on 100 species containing the highest numbers of strains in our dataset (Data S1). Generally, the higher the number of species included in the analysis, the easier it is to distinguish between different strains. The top 100 species were selected to reflect the scenario where both hardly distinguishable collections of strains, such as those comprising *E. coli* and *S. enterica *, and less challenging datasets are present. We considered a strain to be identifiable if at least a single copy of the near-16S and 16S rRNA region differs from all other analogous regions of the same species. We observed that the inclusion of near-16S regions statistically significantly (*P*=1.5e^−11^, one-sided Wilcoxon signed rank test) increases the fraction of identifiable strains as compared to individual hypervariable regions and the full-length gene (Fig. 3e).

Inclusion of near-16S regions increased the classification accuracy at the species level when strains were left out (Fig. 3c). The accuracy increased with increasing length of included genomic fragments up to 400 bp. The mean classification accuracy using 16S rRNA sequences alone was comparable to that of near-16S regions linked to V1–V2. For individual hypervariable regions, the classification accuracy was substantially lower as compared to both full-length gene and near-16S sequences linked to V1–V2.

We tested whether inclusion of near-16S regions improves sequence clustering into OTUs. Inclusion of near-16S regions does not consistently increase any OTU clustering metric (Table S1). As noted earlier [5], thresholds for clustering differ for different datasets, and no clustering value seems to be universal. This is true for both the 16S rRNA and near-16S sequences. In addition, the utility of the 16S rRNA upstream genomic region for clustering into OTUs is limited by high variation of the clustering threshold value depending on metric. For ‘representative’ sequence sets, bijection and Matthews’ correlation coefficient thresholds were approximately two-fold lower than those of richness ratio, and thus if near-16S sequences are included in analysis the choice between clustering accuracy and correct community richness representation needs to be made.

It is increasingly recognized that the relationship between conventional OTUs and real species is largely groundless, and thus exact sequence variants were suggested to improve resolution [40]. However, such sub-OTU analysis suffers from artefactual sequence inaccuracies, which may be treated as non-existent taxonomic units. The overall similarity of 16S rRNA gene sequences makes it complicated to distinguish between real SNP and PCR errors. To test whether sequence differences in the 16S rRNA upstream region would account for PCR errors, we measured the distance between sequence variants by the number of substitutions or indels. For 1000 random sequences, distances were calculated to the five closest non-identical (distance >0) sequences in the dataset. We observed that for 16S rRNA within-gene regions, the distance to the closest sequence is small (1–3 nt) whereas the near-16S region demonstrated a larger distance, especially when the length of the genomic region included in the analysis exceeded 200 bp (Fig. S3). This result indicates that the near-16S region can effectively improve artefactual error detection.

### Semi-targeted sequencing enables capture of *a priori* unknown sequences adjacent to the target site

**Fig. 4.**
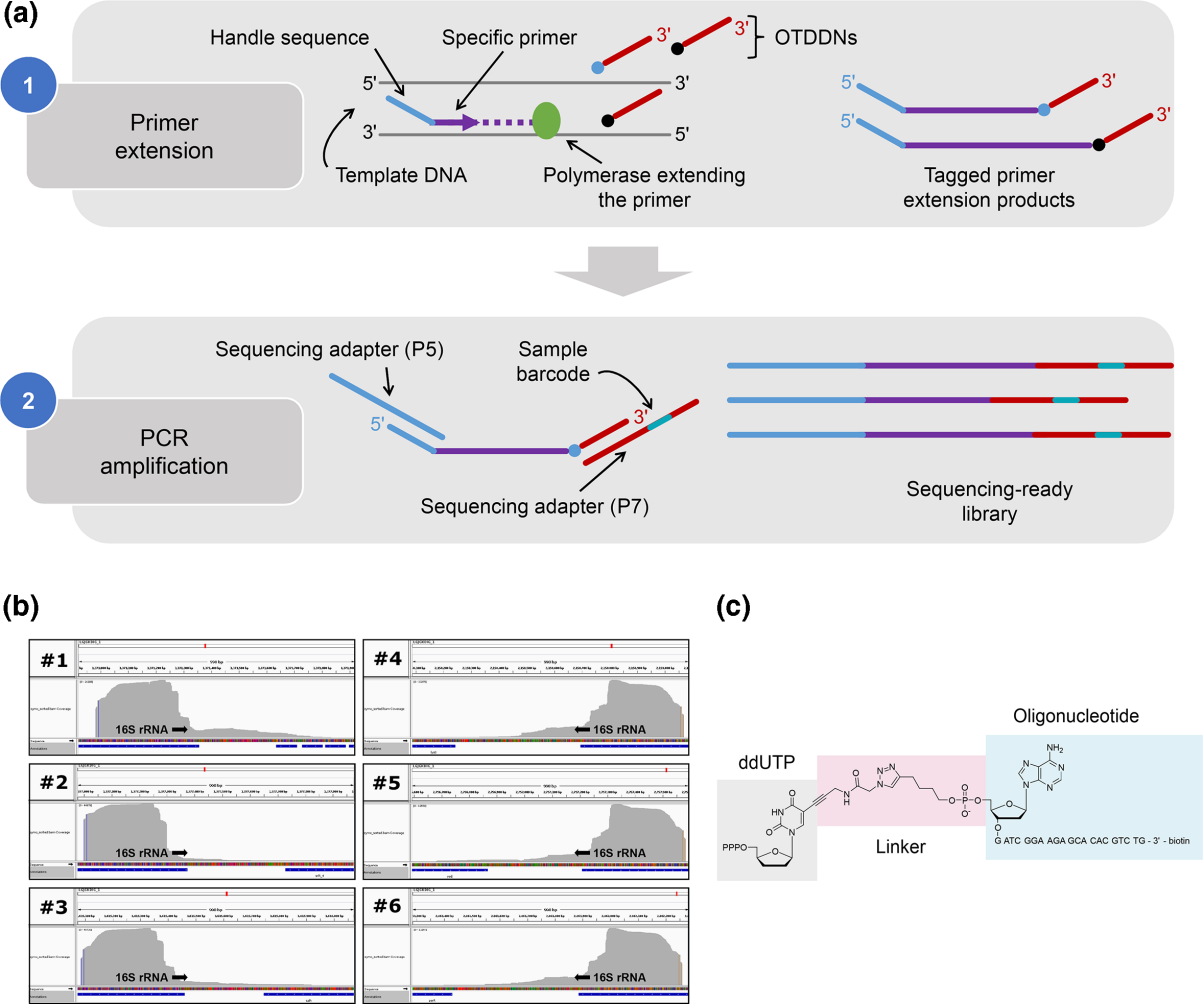
Semi-targeted sequencing approach. (a) Outline of st16S-seq library preparation. (**b**) Read coverage of each of the six 16S rRNA gene copies and upstream regions within the *Listeria monocytogenes* genome. (**c**) The structure of oligonucleotide-tethered dideoxynucleotides (OTDDNs) as exemplified by oligo-modified ddUTP.

To implement the linking of the near-16S region to sequences of the 16S rRNA gene, we developed st16S-seq (Fig. 4a), which utilizes a nucleotide-mediated adapter addition technology for rapid and simple preparation of sequencing-ready molecules by the extension of a single site-specific primer. Specific primers designed for the bacterial st16S-seq target region between V2 and V3 and are oriented towards 16S rRNA upstream sequence. In addition, primers contain universal PCR handles for further library amplification. Upon annealing, primers are extended by polymerase able to incorporate dideoxynucleotides. The nascent DNA strand is terminated by the incorporation of base-modified dideoxynucleotides conjugated to an oligonucleotide. This step fulfils two library preparation requirements at once: the fragmentation step is integrated into the workflow as the average fragment length may be controlled by the ratio of oligonucleotide-tethered dideoxynucleotides (OTDDNs) to corresponding dNTPs, and the obtained fragments are readily labelled by platform-specific adapters at both termini. The resulting extension products are amplified via PCR and subjected to standard Illumina paired-end sequencing. The forward sequencing read (R1) contains 16S rRNA V1–V2 regions starting from the specific priming site while the reverse read (R2) consists of genomic regions upstream the 16S rRNA gene starting from random positions. Reads mapped to six 16S rRNA gene copies and their upstream regions within the *Listeria monocytogenes* genome are shown in Fig. 4(b.

The successful use of ‘click’ chemistry as a means of adapter addition was previously reported [41, 42]. Here, we executed ‘click’ reactions to generate oligo-modified nucleotides with oligonucleotide attached via the 5′ terminus prior to their incorporation into the growing DNA strand (Fig. 4c and S4, see Methods). We obtained conjugates of correct mass and 98% purity with > 30 % yield. An essential requirement for these compounds is the compatibility of the unnatural triazole-based linker with DNA polymerases to enable the use of the attached oligo as a priming site. We optimized the structure of linker and identified polymerases of types A, B, X and RT able to use OTDDNs as substrates as well as polymerases able to perform read-through (manuscript in preparation). These findings pave the way for straightforward DNA labelling by any desired oligonucleotide irrespective of the sequence context of the template.

### st16S-seq accurately estimates microbial composition in low-complexity samples

To assess the performance of st16S-seq, we sequenced ZymoBIOMICS and ATCC microbial community DNA standards, two replicates each. The results of *in silico* analysis suggested the optimal total insert length of 1 kb, although we generated st16S-seq libraries with a median insert size of ~600 bp to comply with the supported range of insert sizes for short-read Illumina sequencers. Nevertheless, the assembly of resulting reads generated genome-linked contigs ranging in size from ~600 to 1200 bp (Fig. S5). Genomic sequences enabled unambiguous identification of intragenomic 16S rRNA gene copies (Fig. 4b) and subsequent correction of read abundances for accurate quantitative estimation of taxa on the sole basis of sequencing data, assuming no prior knowledge of 16S rRNA gene copy numbers. The resulting st16S-seq data strongly correlated with the expected abundance distribution in both analysed mock communities, with Pearson’s correlation coefficients of 0.96–0.97 and 0.88–0.90 for ZymoBIOMICS (Fig. 2a) and ATCC (Fig. 2b) standards, respectively. In contrast, PCR-based techniques demonstrated poorer (Quick-16S and NEXTFLEX V1–V3) or inconsistent (EMP, NEXTFLEX V4, QIAseq and Swift) performance as compared to st16S-seq, which is attributable to widely acknowledged limitations posed by PCR primer design and unequal discriminatory power of individual variable regions [20, 43, 44]. st16S-seq was able to identify the majority of 16S rRNA gene copies in the members of the ZymoBIOMICS mock community (Fig. 2c). Occasionally, one or several copies may remain unresolved because intragenomic sequence differences occur at a marginal distance, which can be captured and reliably sequenced by our technique using short reads. In bacteria with multiple 16S rRNA gene copies, the evolution of 16S rRNA genes is thought to occur not only by vertical transmission of mutations, but also by non-reciprocal recombination with either horizontally acquired or intragenomic donors [45, 46]. Intragenomic recombination events might in turn result in duplications of the chromosomal regions nearby, bringing more complexity to the identification of gene copy numbers.

As we have predicted from the *in silico* analysis, the inclusion of near-16S sequences into the sequencing library improved the classification accuracy at the species level. We have assessed the ability of targeted methods and st16S-seq to discern bacterial species within mock communities employing both unmerged and merged paired-end reads for the analysis. The use of unmerged reads placed st16S-seq on a par with V1–V2-containing amplicons (Quick-16S and NEXTFLEX V1-V3) when analysing 20 species within ATCC microbial standards. Neither sequencing method was able to recognize *Staphylococcus epidermidis* and *Streptococcus agalactiae* from the ATCC mock community with such an analysis strategy. Of eight bacterial species constituting the ZymoBIOMICS mock community, *Bacillus subtilis* was incorrectly annotated by st16S-seq as *Bacillus velezensis *; the same misassignment was observed in V4 datasets (Table S2). In contrast, the analysis of assembled genome-linked contigs in st16S-seq datasets allowed us to correctly identify all members of mock communities, except for *Streptococcus agalactiae *, while the precision of PCR-based methods did not improve from the use of merged reads (Fig. 2d, Table S3). This highlights the importance of bridging 16S rRNA sequences with adjacent genomic regions for the precision of st16S-seq.

### st16S-seq approximates relative abundances obtainable by WGS in high-complexity samples

To analyse the utility of st16S-seq for the characterization of highly complex communities, we sequenced libraries prepared from soil-derived DNA. Highly heterogenous samples exposed the inherent limitation of st16S-seq related to limited ability to assemble genome-linked contigs when individual 16S rRNA gene copies are sparsely covered. Nonetheless, we used unmerged read pairs to perform species-level characterization of soil communities. Alpha diversity within soil samples measured by Shannon's index was substantially higher in whole metagenome sequencing datasets as compared to targeted approaches as well as st16S-seq (Fig. 1a). This observation is not surprising considering < 1 % sequence duplication level in WGS datasets. Among targeted sequencing methods, st16S-seq along with the Quick-16S kit were able to detect the highest bacterial species diversity in all samples. Use of the V4 region resulted in underestimation of species-level abundance. The overlap between species identified by each individual method and jointly by all studied techniques or by WGS alone is given in Fig. 4(b). As the median number of unique on-target reads, which reflects the complexity of the sequenced samples, in st16S-seq datasets was ~10 000, the lowest abundance that could be measured by st16S-seq was approximately 1e10^−4^. Applying an equivalent or higher abundance thresholds demonstrated the superior ability of st16S-seq to detect species overlapping with all other methods, including WGS. The detailed analysis of overlaps between identified bacterial species in each of the soil samples is provided in Figs S6 and S7.

Principal component analysis (PCA) considering the relative abundances of reads assigned per bacterial species across soil samples sequenced using different methods revealed that data based on V4 and NEXTFLEX V1–V3 amplicon sequencing form distinct clusters, while st16S-seq and Quick-16S can approximate the variability of read fractions detected by WGS. Moreover, when st16S-seq data are analysed as pseudocontigs, meaning that all reads associated with individual OTUs are analysed as a whole, st16S-seq clusters with WGS even better (Fig. 1c).

We and others [14, 47] have observed that sequence conservation level of the V1–V2 region is the lowest among within-gene 16S rRNA sequences, suggesting that this sub-region should be of greatest diagnostic power. Practical examples, however, often report underperformance of V1–V2 [48, 49]. It is apparent that PCR-based methods are often unable to make full use of sequence diversity present in that region due to not universally applicable design of commonly used primers. st16S-seq relieves this constraint by capturing variable sequences in a context-independent manner. We looked into the opportunity to exploit only the V1–V2 region in st16S-seq data for species-level characterization. The captured microbial diversity was in line with other targeted techniques (Fig. S8). We argue that the stable performance of st16S-seq enables its use both (i) as a method offering high taxonomic resolution and enabling precise determination of 16S rRNA gene copy numbers, when sample complexity and sequencing depth allow us to assemble genome-linked contigs, and (ii) as a well-performing method to sequence the V1–V2 16S rRNA sub-region without the need to design a primer spanning the highly variable V1 locus.

## Discussion

Here, we have demonstrated that direct linking of near-16S genomic sequences to those of the V1–V2 16S rRNA region gives a number of significant methodological advancements to microbiome characterization. Although alternative approaches to interrogate microbiomes have been suggested [50, 51], the amount of 16S rRNA sequences in the databases greatly exceeds those of other bacterial genes and whole genomes, favouring the use of 16S rRNA as a phylogenetic marker. Considerable effort has been devoted to improving its taxonomic resolution [13, 14], but because of limited diversity of within-gene sequences and hardly predictable gene copy numbers [52] unequivocal characterization of microbiomes was barely possible. st16S-seq solves this by providing a means of establishing a direct connection between 16S rRNA sequences and adjacent genomic regions, which we showed to be highly variable and of high diagnostic value. A similar approach was proposed previously [53] and showed promising results, although low efficiency and an elaborate setup impeded its wider adoption. We have developed an elegant approach of capturing unknown sequences near a defined target site. Semi-targeted library preparation is based on primer extension reaction in the presence of oligonucleotide-tethered chain terminators which ensure stochastic fragmentation and adapter addition at the same time. This sequencing design is modular: primers can be designed to target any gene of interest and oligonucleotide modification conjugated to dideoxynucleotides can also be of any desired sequence, meaning that the same principle might be adapted for a plethora of applications where highly variable regions are adjacent to defined loci. Moreover, sequencing can be conducted on any platform.

Linking 16S rRNA sequences to genomic loci appeared to be a superior strategy for microbiome characterization as compared to individual variable regions and full-length gene sequence, especially when the length of captured genomic region exceeds 200 bp. Admitting that 16S rRNA gene sequences will never be able to represent bacterial species diversity perfectly [47], whole metagenome sequencing is viewed as an alternative to 16S rRNA sequencing providing more precise taxonomic resolution [51]. Nonetheless, the analysis of whole metagenome sequencing data relies heavily on reference databases and requires expansion of reference genomes to cover novel environments. st16S-seq is capable of genome-linked contig assembly in a reference-free manner. Moreover, this approach can assist in metagenomic assembly by accurate location of multiple 16S rRNA copies and serves to establish a consensus classification.

We applied st16S-seq to discern bacterial species in low- and high-complexity samples. The importance of validating any microbiome characterization technique on test samples with known ground truth cannot be underestimated – each step of the sample preparation procedure is a potential source of bias [54, 55]. Here we demonstrated that, thanks to unique design and carefully selected target-specific primers, st16S-seq determines the composition of two different mock microbial communities with very high precision in terms of both classification accuracy and abundance estimation. Soil microbial communities contain the highest level of prokaryotic diversity of any environment [56], thus making it a challenging sample type for any technique. It appeared that ultra-high diversity requires higher sequencing depths to enable genome-linked contig assembly in st16S-seq. Nevertheless, the analysis of unmerged reads and pseudocontigs still provided an adequate approximation of species abundance as compared to that obtainable by WGS.

We thoroughly benchmarked st16S-seq against other commercially available PCR-based microbiome characterization techniques. In our study, amplicons containing V1–V2 or V1–V3 regions exhibited better performance in terms of species-level classification accuracy and better captured alpha diversity in soil samples than those consisting of V4 sequences. In other study involving soil communities [44] it was observed that V4–V5 domain data clustered separately from all other analysed 16S rRNA regions in soil samples, indicating that the V4–V5 domain was skewed regarding the detection of certain phyla. Our results showed that soil data derived from V4-containing amplicons likewise tended to group together, although we also observed that the V1–V3 amplicon formed a distinct cluster. Only st16S-seq and V1–V2 amplicon datasets clustered along with WGS. Sequencing long-range PCR products, spanning the full-length 16S rRNA gene [14, 57] or 16S–23S rRNA region [58], by either short-read or long-read technologies was reported to improve the diagnostic yield in clinical samples. While the rationale to include long-range information lies in capturing greater sequence differences, these techniques are still vulnerable to biases typical for amplicons. Given that st16S-seq requires only one target-specific primer, it is reasonable to believe that the robust performance of st16S-seq would depend to a lesser extent on the application and source of bacterial community compared with PCR primers [20].

Together, st16S-seq enables high-throughput microbiome profiling with unprecedented precision at a cost of targeted sequencing. Further development of st16S-seq could include the combination of semi-targeted library preparation techniques with the long-read sequencing platforms to streamline the assembly of genome-linked contigs.

## Supplementary Data

Supplementary material 1

Supplementary material 2
