# Supplementary material for: High-resolution microbiome analysis enabled by linking of 16S rRNA gene sequences with adjacent genomic contexts
Source: Microb Genom. 2021 Sep 2;7(9):000624. doi: 10.1099/mgen.0.000624 (PMC8715429; doi:10.1099/mgen.0.000624)
Supplement: Supplementary material 1 [file mgen-7-0624-s001.pdf]

Supplementary Figure 1

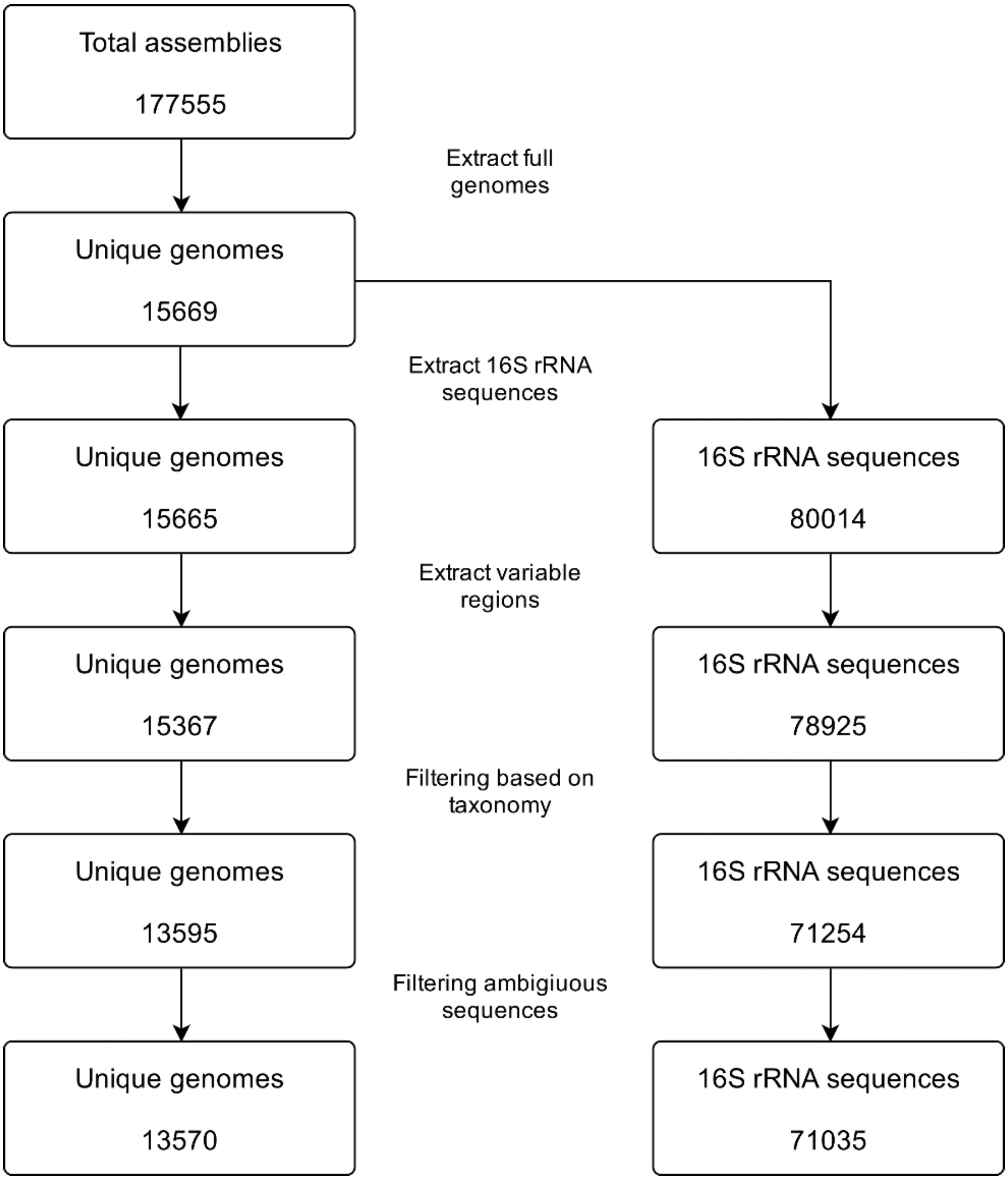

Figure 1. Database preparation and filtering steps with numerical changes of sequences and genomes after every step.

Supplementary Figure 2

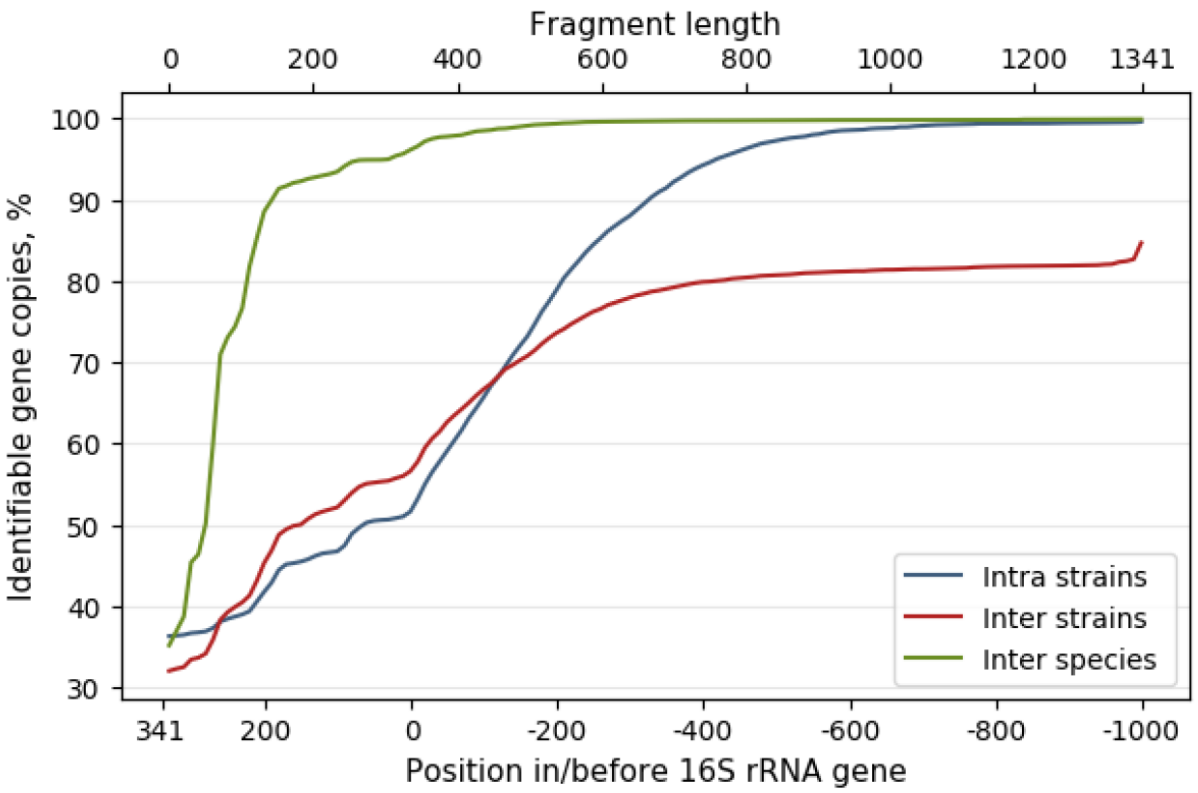

Figure 2. The dependency of 16S rRNA gene copy number identifiability on the analyzed fragment length. The total length of analyzed fragment is shown on the upper X axis. The position in reference *E. coli* 16S rRNA sequence is shown on the bottom X axis.

Supplementary Figure 3

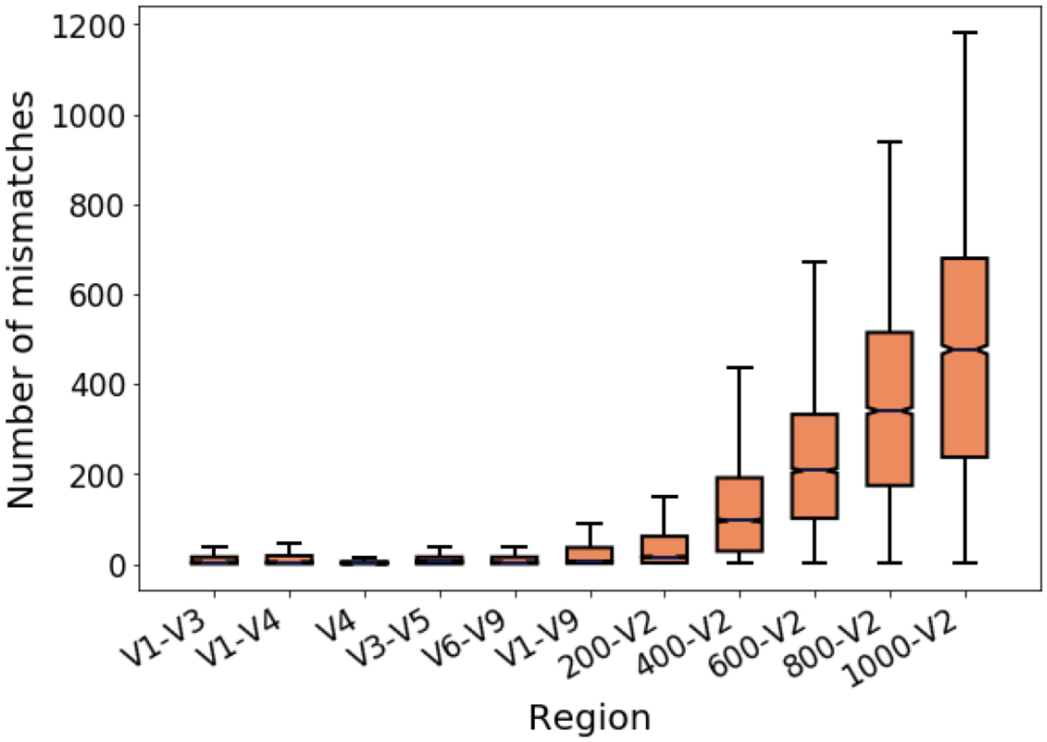

Figure 3. The distribution of sequence mismatch numbers. For near-16S regions the length of genomic fragment (200 bp, 400 bp, 600 bp, 800 bp, 1,000 bp) is indicated. In all cases near-16S region is linked with V1-V2 16S rRNA sequences. Center line – median, box limits – upper and lower quartiles, whiskers – 1.5× interquartile range.

Supplementary Figure 4

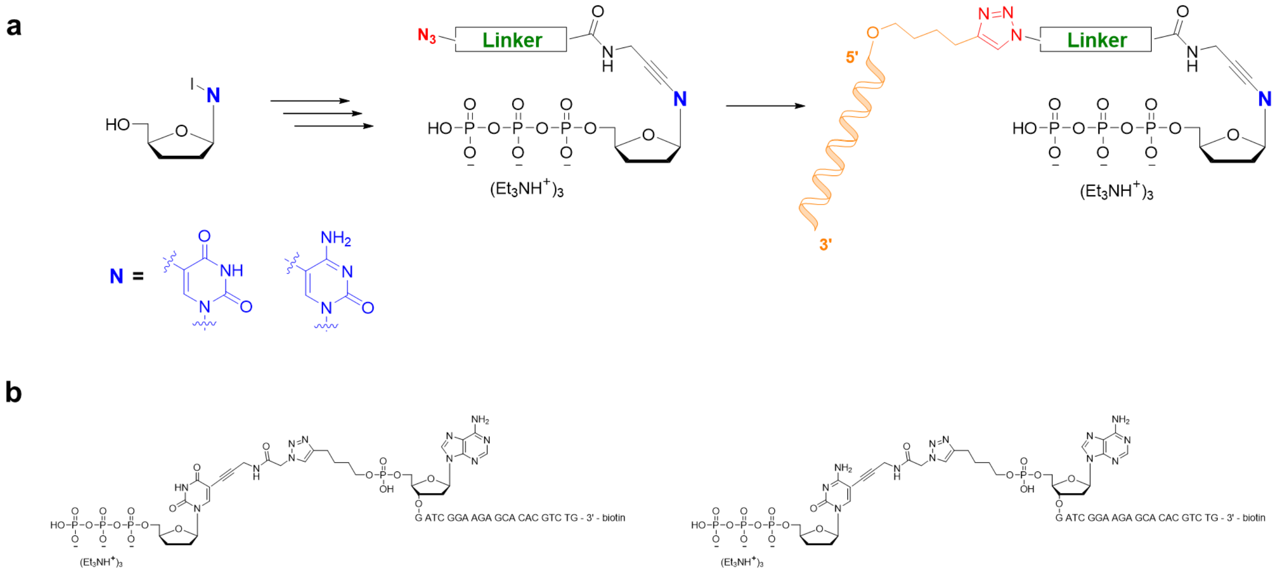

Figure 4. The synthesis of oligonucleotide-tethered dideoxynucleotides. a, The principle of oligonucleotide coupling to azido-modified dideoxynucleotides. **b,** The structure of oligonucleotide-tethered dideoxynucleotides used in this study.

Supplementary Figure 5

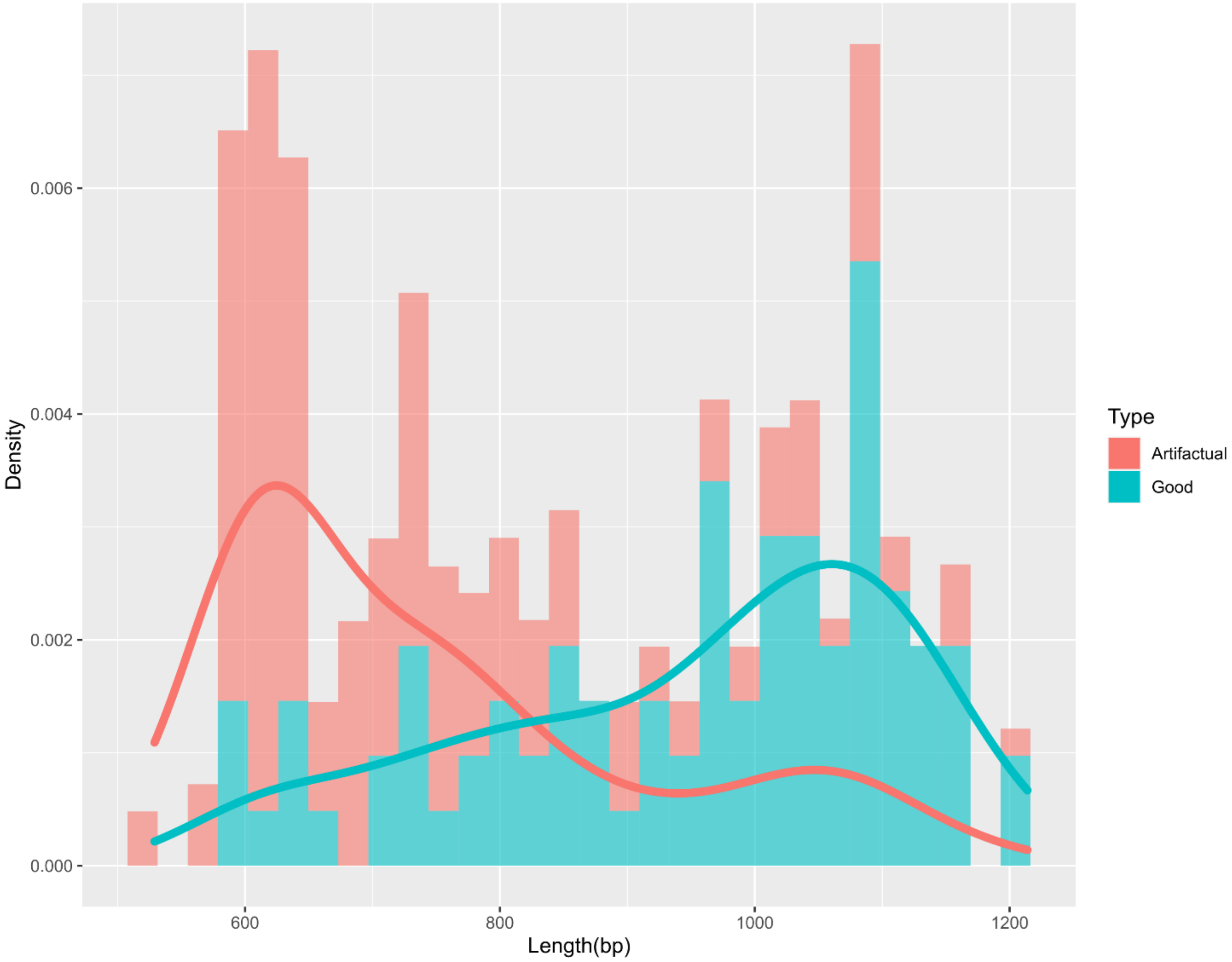

Figure 5. The assembly of genome-linked contigs in st16S-seq data. The size distribution of correct and artifactual contigs assembled in st16S-seq datasets obtained for ZymoBIOMICS mock community.

Supplementary Figure 6

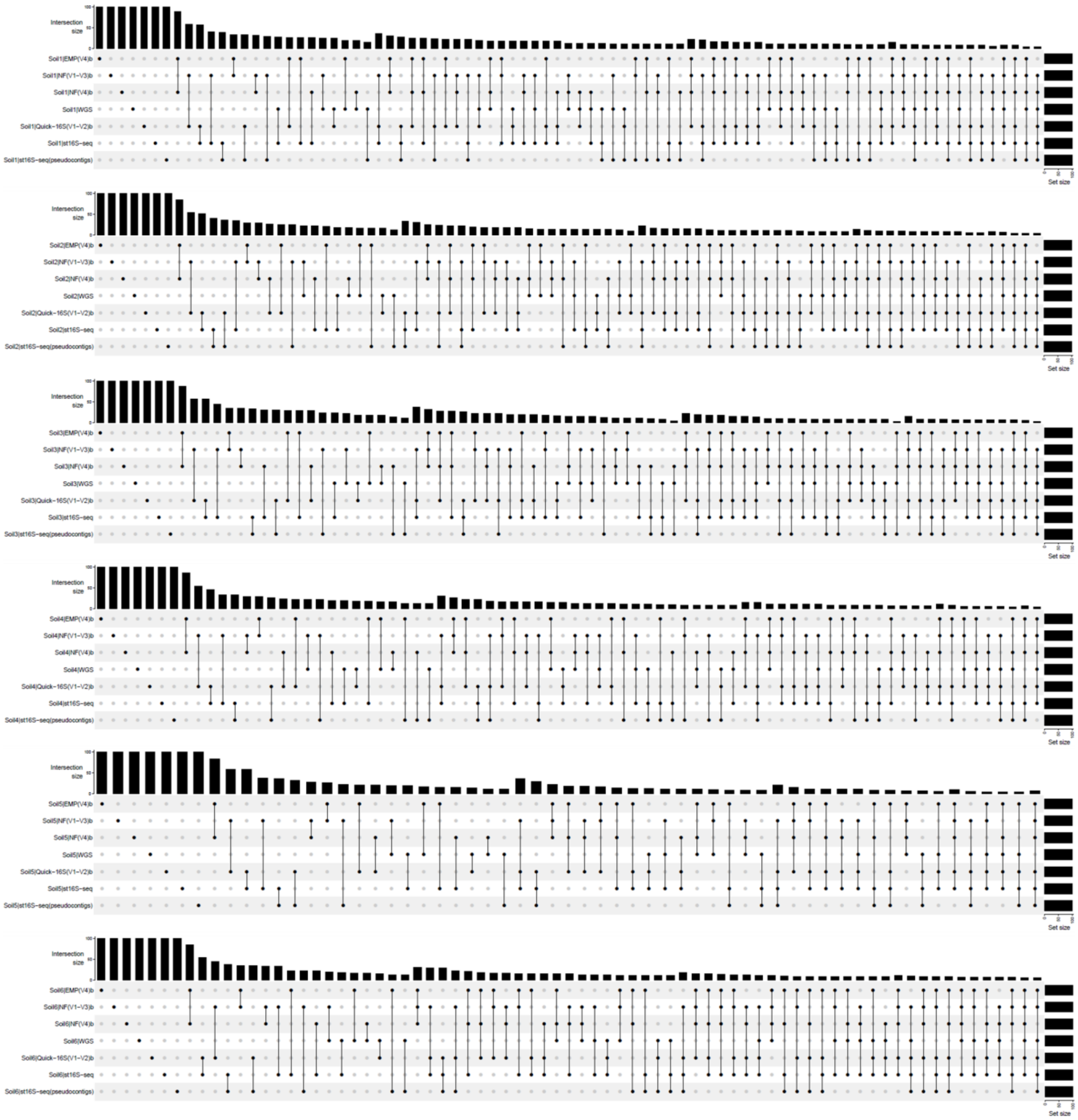

Figure 6. The overlap between the most abundant bacterial species identified within soil datasets generated with st16S-seq and conventional techniques. Each UpSet plot represents intersections of 100 most abundant bacterial species identified in each of the six sequenced soil samples. The list of species and abundance scores is provided in Supplementary Data 2.

Supplementary Figure 7

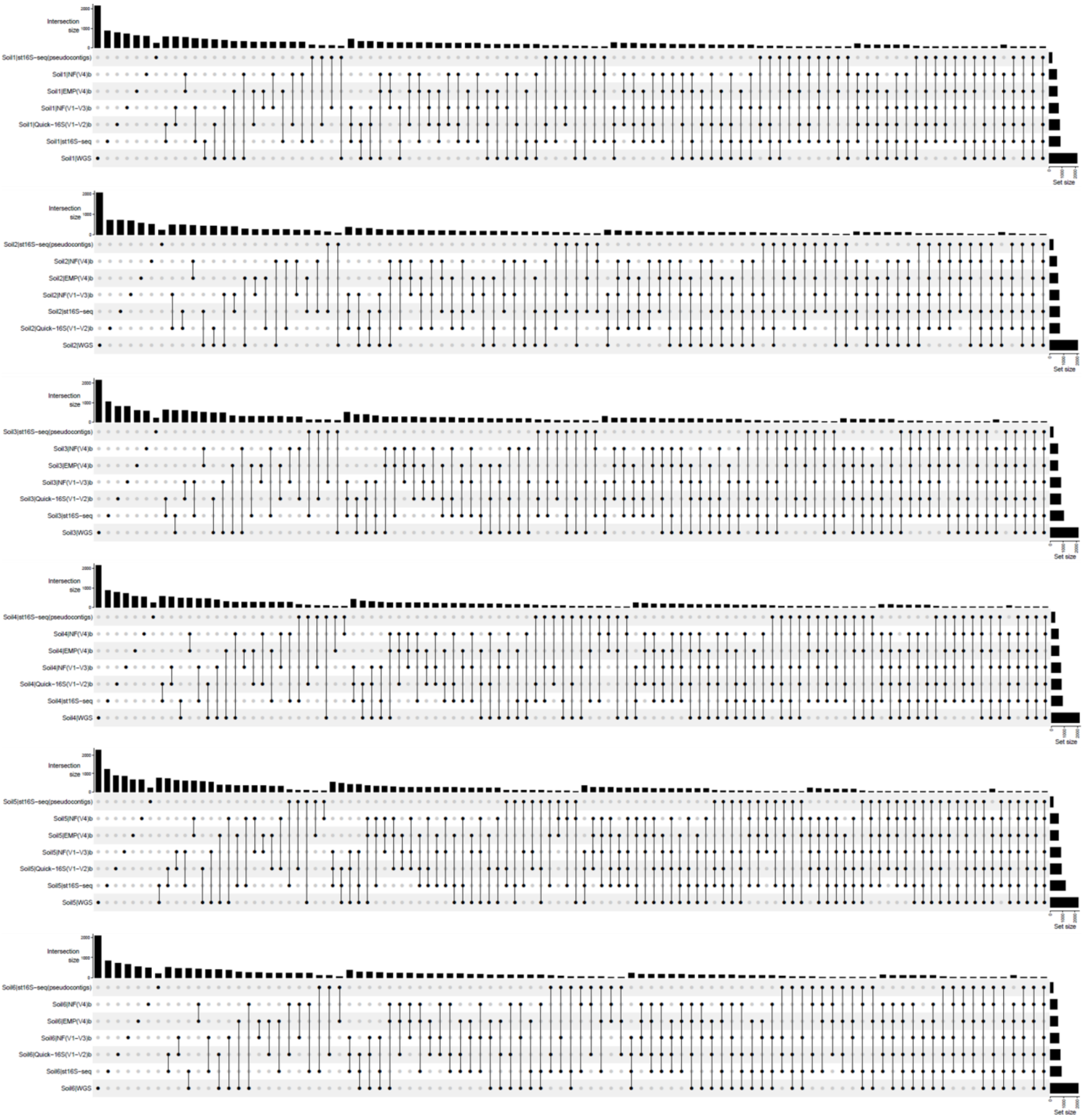

Figure 7. The overlap between bacterial species identified with abundance threshold of 1e-04 within soil datasets generated with st16S-seq and conventional techniques. Each UpSet plot represents intersections of bacterial species identified in each of the six sequenced soil samples. The list of species and abundance scores is provided in Supplementary Data 3.

Supplementary Figure 8

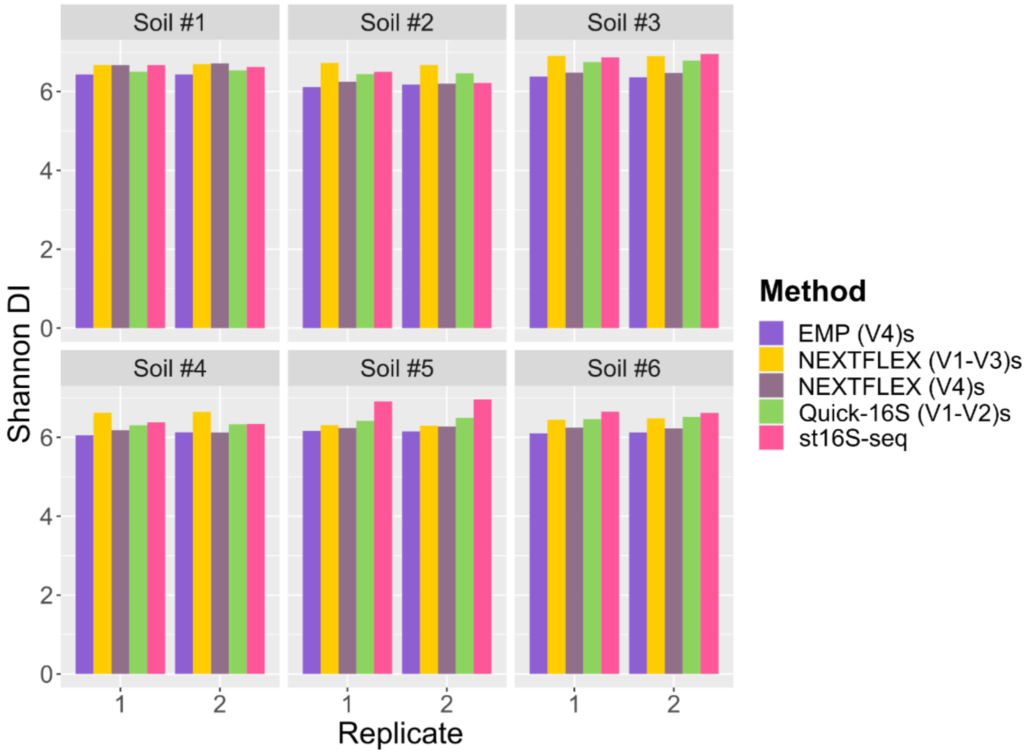

Figure 8. The possibility to use st16S-seq data for OTU-based characterization. Shannon diversity indices for st16S-seq datasets and other amplicon sequencing methods were calculated only on the basis of OTU clustering.

Supplementary Figure 9

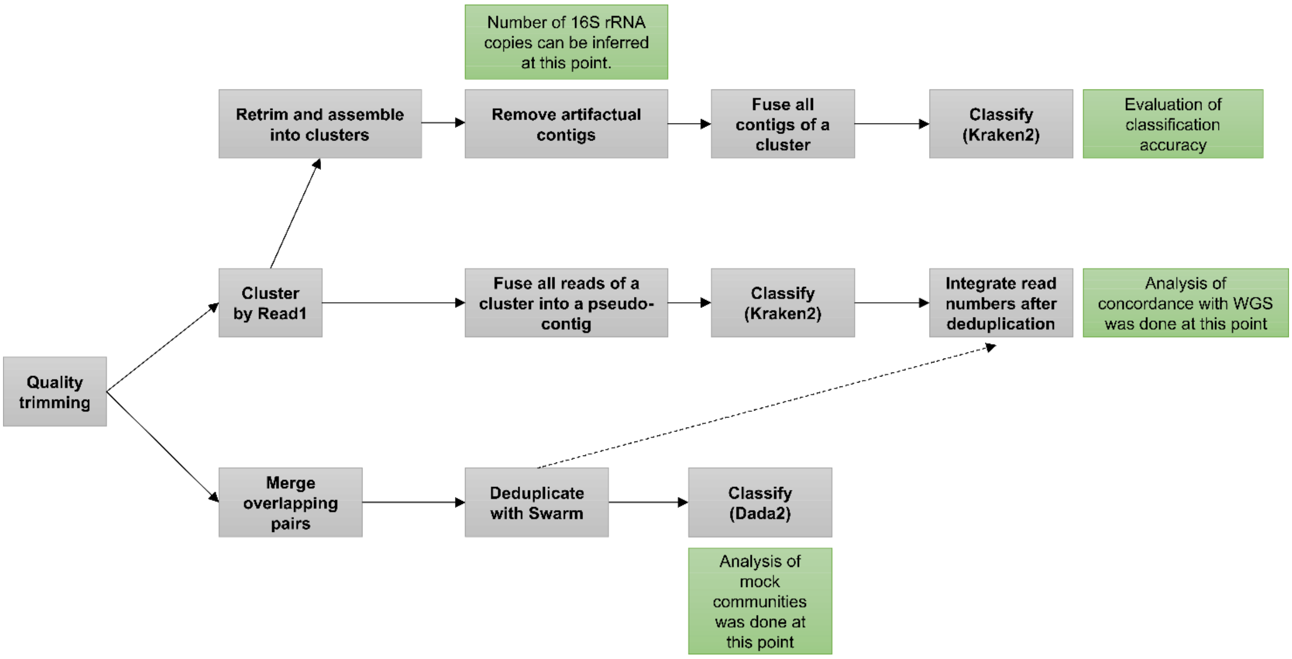

Figure 9. The outline of the st16S-seq data analysis workflow.

Supplementary Table 1

Table 1. Highest clustering metrics obtained for each region and clustering thresholds representing the highest metric. For near-16S sequences the length of genomic fragment used for analysis is indicated (500 bp, 400 bp, 300 bp, 200 bp, 100 bp). In all cases, genomic regions are linked to V1-V2 16S rRNA sequences. Bij – bijection, MCC – Matthews′ Correlation Coefficient, RR – richness ratio, NMI – normalized mutual information.

		**Metric value**				**Clustering threshold**			
**Set**	**Region**	**Bij**	**MCC**	**RR**	**NMI**	**Bij**	**MCC**	**RR**	**NMI**
**All sequences**	**500-V2**	0.519	0.724	0.982	0.921	0.031	0.042	0.065	0.052
	**400-V2**	0.524	0.715	0.997	0.920	0.028	0.056	0.062	0.040
	**300-V2**	0.523	0.724	0.997	0.919	0.027	0.066	0.056	0.065
	**200-V2**	0.538	0.832	0.995	0.926	0.020	0.059	0.048	0.055
	**100-V2**	0.556	0.706	0.999	0.921	0.017	0.049	0.038	0.049
	**V1-V2**	0.562	0.722	0.997	0.925	0.013	0.029	0.030	0.029
	**V1-V3**	0.572	0.764	0.996	0.926	0.011	0.037	0.033	0.037
	**V3-V5**	0.565	0.863	0.994	0.931	0.004	0.020	0.019	0.015
	**V4**	0.493	0.833	0.996	0.923	0.003	0.008	0.008	0.009
	**V6-V9**	0.548	0.567	0.999	0.907	0.005	0.015	0.017	0.015
	**V1-V9**	0.571	0.668	0.990	0.910	0.007	0.030	0.031	0.030
**Representative sequences**	**500-V2**	0.597	0.645	0.995	0.965	0.020	0.013	0.039	0.018
	**400-V2**	0.602	0.655	1.000	0.966	0.018	0.013	0.036	0.018
	**300-V2**	0.608	0.656	0.994	0.966	0.016	0.013	0.032	0.016
	**200-V2**	0.610	0.646	0.996	0.966	0.018	0.011	0.028	0.016
	**100-V2**	0.623	0.649	0.997	0.967	0.014	0.011	0.020	0.014
	**V1-V2**	0.625	0.621	0.998	0.967	0.013	0.009	0.013	0.013
	**V1-V3**	0.655	0.695	0.995	0.972	0.009	0.009	0.014	0.009
	**V3-V5**	0.638	0.615	0.985	0.968	0.004	0.002	0.004	0.004
	**V4**	0.555	0.492	0.966	0.954	0.000	0.000	0.000	0.000
	**V6-V9**	0.622	0.661	0.999	0.967	0.005	0.005	0.007	0.005
	**V1-V9**	0.657	0.717	0.990	0.972	0.006	0.005	0.012	0.006

Supplementary Table 2

Table 2. Species-level characterization of mock microbial communities using NCBI database as a reference and unmerged read pairs as an input for Kraken. In case of st16S-seq, no genome-linked contigs were assembled prior to species identification.

Correctly identified species are marked with a dot, and in case of incorrect identification, actually identified taxon is given.

Group	Expected species	EMP (V4)	NEXTFLEX (V1-V3)	NEXTFLEX (V4)	Quick-16S (V1-V2)	st16S-seq
**ATCC**	*Acinetobacter baumannii*	*Acinetobacter pittii*	•	*Acinetobacter pittii*	•	•
	Bacillus cereus	*Bacillus velezensis*	•	*Bacillus thuringiensis*	•	•
	Bifidobacterium adolescentis	*Bifidobacterium longum*	•	*Bifidobacterium longum*	•	•
	Clostridium beijerinckii	*Clostridium butyricum*	•	*Clostridium butyricum*	•	•
	Cutibacterium acnes	•	•	•	•	•
	Deinococcus radiodurans	•	•	•	•	•
	Enterococcus faecalis	*Enterococcus faecium*	•	*Enterococcus faecium*	•	•
	Escherichia coli	•	•	•	•	•
	Helicobacter pylori	•	•	•	•	•
	Lactobacillus gasseri	*Lactobacillus delbrueckii*	•	*Lactobacillus delbrueckii*	•	*Lactobacillus helveticus*
	Neisseria meningitidis	•	•	•	•	•
	Bacteroides vulgatus	•	•	•	•	•
	Porphyromonas gingivalis	•	•	•	•	•
	Pseudomonas aeruginosa	•	•	•	•	•
	Rhodobacter sphaeroides	•	•	•	•	•
	Schaalia odontolytica	•	•	•	•	•
	Staphylococcus aureus	•	•	•	•	•
	Staphylococcus epidermidis	*Staphylococcus pseudintermedius*	*Staphylococcus pseudintermedius*	*Staphylococcus pseudintermedius*	*Staphylococcus pseudintermedius*	*Staphylococcus simiae*
	Streptococcus agalactiae	*Streptococcus dysgalactiae*	*Streptococcus dysgalactiae*	*Streptococcus dysgalactiae*	*Streptococcus dysgalactiae*	*Streptococcus dysgalactiae*
	Streptococcus mutans	•	•	•	•	•
**ZymoBIOMICS**	*Bacillus subtilis*	*Bacillus velezensis*	•	*Bacillus velezensis*	•	*Bacillus velezensis*
	Pseudomonas aeruginosa	•	•	•	•	not identified
	Enterococcus faecalis	*Enterococcus faecium*	•	*Enterococcus faecium*	•	•
	Escherichia coli	•	•	•	•	•
	Lactobacillus fermentum	•	•	•	•	•
	Listeria monocytogenes	•	•	•	•	•
	Salmonella enterica	•	•	•	•	•
	Staphylococcus aureus	*Staphylococcus pseudintermedius*	•	•	•	•

Supplementary Table 3

Table 3. Species-level characterization of mock microbial communities using NCBI database as a reference and merged reads as an input for Kraken. In case of st16S-seq, contig assembly bridging 16S rRNA sequence information with adjacent genomic context was performed prior to species identification.

Correctly identified species are marked with a dot, and in case of incorrect identification, actually identified taxon is given.

	Expected species	EMP (V4)	NEXTFLEX (V1-V3)	NEXTFLEX (V4)	Quick-16S (V1-V2)	st16S-seq (contigs)
**ATCC**	*Acinetobacter baumannii*	*Acinetobacter pittii*	*Acinetobacter venetianus*	*Acinetobacter pittii*	•	•
	Bacillus cereus	*Bacillus velezensis*	•	*Bacillus thuringiensis*	•	•
	Bifidobacterium adolescentis	*Bifidobacterium longum*	•	*Bifidobacterium longum*	•	•
	Clostridium beijerinckii	*Clostridium butyricum*	•	*Clostridium butyricum*	•	•
	Cutibacterium acnes	•	•	•	•	•
	Deinococcus radiodurans	•	•	•	•	•
	Enterococcus faecalis	*Enterococcus faecium*	•	*Enterococcus faecium*	•	•
	Escherichia coli	•	•	•	•	•
	Helicobacter pylori	•	•	•	•	•
	Lactobacillus gasseri	*Lactobacillus delbrueckii*	•	*Lactobacillus delbrueckii*	•	•
	Neisseria meningitidis	•	•	•	•	•
	Bacteroides vulgatus	•	•	•	•	•
	Porphyromonas gingivalis	•	•	•	•	•
	Pseudomonas aeruginosa	•	•	•	•	•
	Rhodobacter sphaeroides	•	•	•	•	•
	Schaalia odontolytica	•	•	•	•	•
	Staphylococcus aureus	*Staphylococcus pseudintermedius*	•	*Staphylococcus pseudintermedius*	•	•
	Staphylococcus epidermidis	*Staphylococcus lugdunensis*	•	*Staphylococcus lugdunensis*	*Staphylococcus pseudintermedius*	•
	Streptococcus agalactiae	*Streptococcus dysgalactiae*	*Streptococcus pyogenes*	*Streptococcus dysgalactiae*	*Streptococcus dysgalactiae*	*Streptococcus dysgalactiae*
	Streptococcus mutans	•	•	•	•	•
**ZymoBIOMICS**	*Bacillus subtilis*	*Bacillus velezensis*	•	*Bacillus velezensis*	•	•
	Pseudomonas aeruginosa	•	not identified	•	•	•
	Enterococcus faecalis	*Enterococcus faecium*	•	*Enterococcus faecium*	•	•
	Escherichia coli	•	•	•	•	•
	Lactobacillus fermentum	•	•	•	•	•
	Listeria monocytogenes	•	•	•	•	•
	Salmonella enterica	•	•	•	•	•
	Staphylococcus aureus	*Staphylococcus pseudintermedius*	•	*Staphylococcus pseudintermedius*	•	•

Supplementary Table 4

Table 4. Locations and specifications of primers used to extract variable region positions in 16S rRNA gene. (a) – conventional oligo names; (b) – oligo names used in dataset built in this study; (c) – coverage across species with no mismatches allowed; (d) – coverage across species with one mismatch allowed; (e) – hypothetical region defined for convenience.

Oligo name[(a)]	Oligo name[(b)]	Direction	Sequence (5′-3′)	Position	Coverage[(c)], %	Coverage[(d)], %
1061F	V7	Reverse	CRRCACGAGCTGACGAC	1061-1077	96.4	98.8
530F	V4	Forward	GTGCCAGCMGCNGCGG	515-530	96.9	98.7
Bakt_341F	V3	Forward	CCTACGGGNGGCWGCAG	341-357	94.9	98.1
909F	V6	Forward	ACTCAAAKGAATWGACGG	909-926	91.7	97.5
802R	V5	Reverse	TACNVGGGTATCTAATCC	785-802	92.5	96.5
GM12R	V8	Reverse	CGTCATCCMCACCTTCCTC	1175-1193	61.4	89.4
1391R	V9	Reverse	GACGGGCGGTGWGTRCA	1391	84.6	87.8
27F	V1	Forward	AGRGTTYGATYMTGGCTCAG	8-27	86.4	92.6
1492R	V10[(e)]	Reverse	TACGGYTACCTTGTTAYGACTT	1470-1492	-	-

Supplementary Table 5

Table 5. Number of mismatches for each 16S rRNA gene primer used to define the corresponding hypervariable region in the dataset built in this study. (a) – hypothetical region defined for convenience.

Mismatch count	Sequence count per hypervariable region								
	**V1**	**V3**	**V4**	**V5**	**V6**	**V7**	**V8**	**V9**	**V10[(a)]**
**0**	79056	79547	79792	79465	78240	79716	64906	79705	72786
**1**	502	298	148	357	1706	249	11763	208	3888
**2**	28	92	20	74	27	10	3166	8	3207
**3**	253	29	10	78	9	4	49	8	12
**4**	30	10	12	15	14	4	4	41	9
**5**	12	9	14	5	3	4	10	15	8
**6**	7	18	7	6	3	0	38	3	3
**7**	25	3	6	2	0	3	61	3	4
**8**	13	4	0	4	4	5	7	10	23
**9**	7	1	1	3	6	3	4	10	8
**10**	14	3	0	3	1	6	5	2	5
**11**	8	0	0	1	0	0	1	0	3
**12**	55	0	0	0	0	0	0	0	9
**13**	0	0	0	0	0	0	0	0	21
**14**	0	0	0	0	0	0	0	0	12
**15**	0	0	0	0	0	0	0	0	6

Supplementary Table 6

Table 6. Primers used in this study. Underlined sequence corresponds to i5 partial Illumina adapter.

Oligo name	Oligo sequence (5′-3′ direction)
PR1	CTCTTTCCCTACACGACGCTCTTCCGATCTTCCCCACTGCTGCCTCCCGTAGGAG
PR2	CTCTTTCCCTACACGACGCTCTTCCGATCTACGCGGCGTCGCTGCATCAGG
PR3	CTCTTTCCCTACACGACGCTCTTCCGATCTGCAAGATTCCCCACTGCTGCCTCCCGTAGG

Supplementary Table 7

Table 7. Commercially available kits for high-throughput 16S rRNA gene sequencing selected for comparative analysis

Kit / Protocol	Cat. No.	Supplier	16S rRNA gene region covered
QIAseq 16S/ITS Screening Panel	333812	Qiagen	V1-V2, V2-V3, V3-V4, V4-V5, V5-V7, V7-V9
Swift Amplicon 16S+ITS Panel	AL-51648	Swift Biosciences	V1-V4, V3-V4, V4-V5, V6-V9, V7-V8
NEXTFLEX 16S V1-V3 Amplicon-Seq Kit for Illumina Platforms	NOVA-4202-02	PerkinElmer	V1-V3
NEXTFLEX 16S V4 Amplicon-Seq Kit 2.0 for Illumina Platforms	NOVA-4203-02	PerkinElmer	V4
Quick-16S NGS Library Prep Kit	D6410	Zymo Research	V1-V2
16S Illumina amplicon protocol	N/A	Earth Microbiome Project[2]	V4
